# Chinese Stroke Association guidelines for clinical management of ischaemic cerebrovascular diseases: executive summary and 2023 update

**DOI:** 10.1136/svn-2023-002998

**Published:** 2023-12-27

**Authors:** Liping Liu, Zixiao Li, Hongyu Zhou, Wanying Duan, Xiaochuan Huo, Weihai Xu, Shujuan Li, Ximing Nie, Huihui Liu, Jinjie Liu, Dapeng Sun, Yufei Wei, Guitao Zhang, Weizhuang Yuan, Lina Zheng, Jingyi Liu, David Wang, Zhongrong Miao, Yongjun Wang

**Affiliations:** 1 Department of Neurology, Beijing Tiantan Hospital, Capital Medical University, Beijing, China; 2 China National Clinical Research Center for Neurological Diseases, Beijing, China; 3 National Center for Healthcare Quality Management in Neurological Diseases, Beijing, China; 4 Research Unit of Artificial Intelligence in Cerebrovascular Disease, Chinese Academy of Medical Sciences, Beijing, China; 5 Chinese Institute for Brain Research, Beijing, China; 6 Neurological Disease Center, Cerebral Vascular Disease Department, Beijing Anzhen Hospital, Capital Medical University, Beijing, China; 7 Department of Neurology, State Key Laboratory of Complex Severe and Rare Diseases, Peking Union Medical College Hospital, Chinese Academy of Medical Sciences and Peking Union Medical College, Beijing, China; 8 Department of Neurology, National Clinical Research Center for Cardiovascular Diseases, Fuwai Hospital, National Center for Cardiovascular Diseases, Chinese Academy of Medical Sciences and Peking Union Medical College, Beijing, China; 9 Department of Neurology and Suzhou Clinical Research Center of Neurological Disease, the Second Affiliated Hospital of Soochow University, Suzhou, China; 10 Department of General Medicine, Dalian Municipal Central Hospital Affiliated Dalian University of Technology, Dalian, China; 11 Interventional Neuroradiology, Department of Neurology, Beijing Tiantan Hospital, Capital Medical University, Beijing, China; 12 Neurovascular Division, Department of Neurology, Barrow Neurological Institute, St. Joseph's Hospital and Medical Center, Phoenix, Arizona, USA; 13 Advanced Innovation Center for Human Brain Protection, Capital Medical University, Beijing, China

**Keywords:** Ischaemic Cerebrovascular Diseases, Guidelines, Management, Diagnosis

## Abstract

**Background:**

China is one of the countries with the highest burden of stroke. Implementing multidimensional management guidelines will help clinicians practise evidence-based care, improve patient outcomes and alleviate societal burdens. This update of the 2019 edition will provide the latest comprehensive recommendations for the diagnosis and treatment of ischaemic cerebrovascular diseases.

**Methods:**

We conducted a comprehensive search on MEDLINE (via PubMed) up to 31 August 2023. The writing team established the recommendations through multiple rounds of online and offline discussions. Each recommendation was graded using the evidence grading algorithm developed by the Chinese Stroke Association (CSA). The draft was reviewed and finalised by the CSA Stroke Guidelines Writing Committee.

**Results:**

This update included revisions of 15 existing recommendations and 136 new recommendations in the following areas of stroke care: emergency assessment and diagnosis of ischaemic cerebrovascular disease, acute-phase reperfusion therapy, evaluation of underlying mechanisms, antithrombotic therapy, prevention and treatment of complications, and risk factor management.

**Conclusions:**

This guideline updated the recommendations for the clinical management of ischaemic cerebrovascular disease from 2019.

## Highlights

The clinical management of ischaemic cerebrovascular diseases comprises a total of 287 recommendations, including 136 new recommendations and 15 revised recommendations from the version in 2019. These highlights reflect significant new therapeutic options developed in recent times that will impact the daily management of patients with acute ischaemic stroke (AIS).

### Reperfusion therapy

1. Tenecteplase (TNK) 0.25 mg/kg intravenous push has been proven non-inferior to intravenous standard dosage of recombinant tissue plasminogen activator (rt-PA) to treat patients with AIS with <4.5 hours of onset (Section 3.1).
2. For patients with anterior circulation large vessel occlusion (LVO) type of AIS who present within 4.5 hours of symptom onset, the efficacy of intravenous TNK (0.25 mg/kg) is non-inferior to intravenous rt-PA (0.9 mg/kg) before intra-arterial (IA) mechanical thrombectomy (MT). The TNK might offer better reperfusion outcomes, while the incidence of symptomatic intracerebral haemorrhage (sICH) remains similar (Section 3.1).
3. For patients with AIS with anterior circulation LVO and a large core infarct within 24 hours of onset and who meet the inclusion criteria of the RESCUE-Japan LIMIT, ANGEL-ASPECT and SELECT 2 trials, IA MT is recommended (Section 3.2).
4. For patients with acute basilar artery occlusion (BAO) within 6 hours of onset who meet the inclusion criteria of the ATTENTION trial, IA MT is recommended (Section 3.2).
5. Patients with acute BAO within 6–12 hours of onset are recommended for IA MT when they meet the inclusion criteria of the ATTENTION or BAOCHE trials (Section 3.2).
6. Patients with acute BAO within 12–24 hours of onset are recommended for MT when they meet the inclusion criteria of the BAOCHE trial (Section 3.2).

### Antiplatelet therapy

1. Intravenous tirofiban can be beneficial in those patients who meet the RESCUE BT2 trial inclusion criteria (Section 4.1).
2. For patients with non-cardioembolic minor ischaemic stroke (IS) (National Institutes of Health Stroke Scale (NIHSS) score ≤3) or high-risk transient ischaemic attack (TIA) (ABCD2 score ≤4) who present within 24 hours of symptom onset, if *CYP2C19* gene testing can be tested and the patient carries *CYP2C19* loss-of-function (LoF) alleles, ticagrelor plus aspirin for 21 days (ticagrelor loading dose of 180 mg on the first day, followed by 90 mg two times per day) and continue with ticagrelor monotherapy (90 mg two times per day) for 90 days are recommended (Section 4.2).
3. For patients with moderate IS (NIHSS score of 4–5) who present within 24 hours of symptom onset, ticagrelor plus aspirin for 30 days (ticagrelor loading dose of 180 mg on the first day, followed by 90 mg two times per day) may reduce the risk of recurrent stroke and death within 30 days (Section 4.2).

### Brain cytoprotection

1. Brain cytoprotection with edaravone dexborneol (intravenous 37.5 mg/dose, once every 12 hours, for 14 days) may improve clinical outcomes in patients with AIS (Section 5.1).
2. DL-3-n-butylphthalide (NBP), 25 mg, dissolved in 100 mL sodium chloride and given as intravenous injection two times per day for the first 14 days, followed by soft 0.2 g capsules of NBP three times a day for the next 76 days, may serve as an adjunct treatment to reperfusion therapy and have the potential to improve functional outcomes in patients with AIS (Section 5.1).

### Risk factor management

1. For patients who cannot tolerate statins or have contraindications to statin therapy, the use of proprotein convertase subtilisin/kexin 9 (PCSK9) inhibitors or ezetimibe may be considered (Section 9.2).
2. For patients with IS or TIA with fasting triglycerides (TG) ≥135 mg/dL (1.52 mmol/L), who have received moderate or high-intensity statin therapy, a glycated haemoglobin (HbA1c) level <10%, and no history of pancreatitis, atrial fibrillation, or severe heart failure, treatment with icosapent ethyl (2 g two times per day) can reduce the risk of stroke recurrence (Section 9.2).

## Introduction

The incidence of stroke in the Chinese population continues to rise, accounting for nearly one-fourth of the global annual stroke cases.^1^ Among adults aged 40 years or above in China, IS accounted for approximately 86.8% of all strokes.^2^ Timely updates to the guidelines can provide new evidence-based recommendations for diagnosis, acute-phase treatment, prevention and management of complications, and secondary prevention for IS.^3^

Since the publication of the 2019 Chinese Stroke Association (CSA) guidelines, notable advancements have emerged in acute-phase reperfusion therapy and antiplatelet treatments for secondary IS prevention. The findings of several high-quality randomised controlled trials (RCTs) conducted in China will have an impact on stroke care.^4–6^ Some new lipid-lowering agents are also helpful in stroke prevention.

We conducted a comprehensive search of MEDLINE (via PubMed) up to 31 August 2023, and compiled the relevant information into a tabular format. The writing team established the level of recommendation through multiple rounds of online and offline discussions. Each recommendation was graded using the evidence grading algorithm developed by the CSA (table 1). The updated guideline kept the nine sections: definitions, emergency assessment and diagnosis, reperfusion therapy, antiplatelet therapy, other treatments in the acute phase, general supportive treatment and management of complications, early evaluation of the aetiology and pathogenesis, interventions targeting aetiology and pathogenesis, risk factor management and long-term intervention.

**Table 1:** The recommended classification and levels of evidence developed by the Chinese Stroke Association

	Class I (benefit>>>risk) The procedure/treatment should be implemented/administered.	Class IIa (benefit>>risk) It is reasonable to conduct specialised research to implement/administer the procedure/treatment with specific objectives.	Class IIb (benefit≥risk) Multiple studies are needed, and more registered data would be helpful. It may be worth considering the implementation/administration of the procedure/treatment.	Class III (risk=benefit or risk>benefit)
Level A				
Multiple diverse populations were assessed. The data are sourced from multiple RCTs or meta-analyses.	The recommended procedure/treatment is beneficial/effective. Multiple RCTs or meta-analyses provide sufficient evidence.	The recommendation leans towards the usefulness/effectiveness of the procedure/treatment. The evidence from multiple RCTs or meta-analyses is inconsistent.	The recommendation regarding effectiveness/efficacy has not been widely recognised. The evidence from multiple RCTs or meta-analyses is highly inconsistent.	The recommended procedure/treatment is not beneficial/ineffective and may even be harmful. Multiple RCTs or meta-analyses provide sufficient evidence.
Level B				
The assessed population was limited. The data are derived from a single RCT or non-randomised studies.	The recommended procedure/treatment is beneficial/effective. The evidence from an RCT or non-randomised studies.	The recommendation leans towards the usefulness/effectiveness of the procedure/treatment. The evidence from a single RCT or non-randomised studies is inconsistent.	The recommendation regarding effectiveness/efficacy has not been widely recognised. The evidence from a single RCT or non-randomised studies is highly inconsistent.	The recommended procedure/treatment is not beneficial/ineffective and may even be harmful. The evidence from an RCT or non-randomised studies.
Level C				
The assessed population was extremely limited. Expert consensus opinions, case studies or diagnostic/treatment guidelines.	The recommended procedure/treatment is beneficial/effective. Expert consensus opinions, case studies or diagnostic/treatment guidelines.	The recommendation leans towards the usefulness/effectiveness of the procedure/treatment. Expert opinions are divergent, case studies or diagnostic/treatment guidelines.	The recommendation regarding effectiveness/efficacy has not been widely recognised. Expert opinions are divergent, case studies or diagnostic/treatment guidelines.	The recommended procedure/treatment is not beneficial/ineffective and may even be harmful. Expert consensus opinions, case studies or diagnostic/treatment guidelines.

RCTs, randomised controlled trials.

### Section 1: definitions associated with ischaemic cerebrovascular diseases

The definitions associated with ischaemic cerebrovascular diseases are shown in table 2.

**Table 2:** Relative definitions of ischaemic cerebrovascular disease

Relative disease	Definition
Ischaemic cerebrovascular disease	It refers to degeneration, necrosis or transient functional loss of local brain tissue, including nerve cells, glial cells and connective fibres, due to vascular obstruction. It is a common clinical disease, predominantly affecting middle-aged and elderly individuals, with high rates of disability and mortality.
Ischaemic stroke	It refers to ischaemic necrosis or softening of local brain tissue caused by cerebral blood circulation disorder, resulting in ischaemia and hypoxia.
Transient ischaemic attack (TIA)	It refers to transient neurological dysfunction caused by focal ischaemia in the brain, spinal cord or retina without acute infarction.
Non-disabling ischaemic cerebrovascular events	It refers to ischaemic cerebrovascular diseases without residual neurological disability, which includes the following three categories: TIA, minor stroke (NIHSS score ≤3 or ≤5), and stroke with rapid resolution and no residual disability. Its clinical features often include mild symptoms at onset or rapid and complete resolution, without leaving any or only mild residual neurological deficits, which do not affect daily life and work.
Disabling ischaemic cerebrovascular events	It refers to ischaemic cerebrovascular events that result in significant residual disabilities after the onset.
Atherosclerotic cardiovascular diseases	It refers to various clinical diseases with ischaemic or endothelial dysfunction–inflammatory changes caused by atherosclerosis, including acute coronary syndrome, myocardial infarction, stable or unstable angina pectoris, post-coronary revascularisation, atherosclerosis-related stroke or TIA, peripheral arterial disease or post-peripheral arterial reconstruction.

NIHSS, National Institutes of Health Stroke Scale.

### Section 2: emergency assessment and diagnosis of patients with AIS

**Figure 1:**
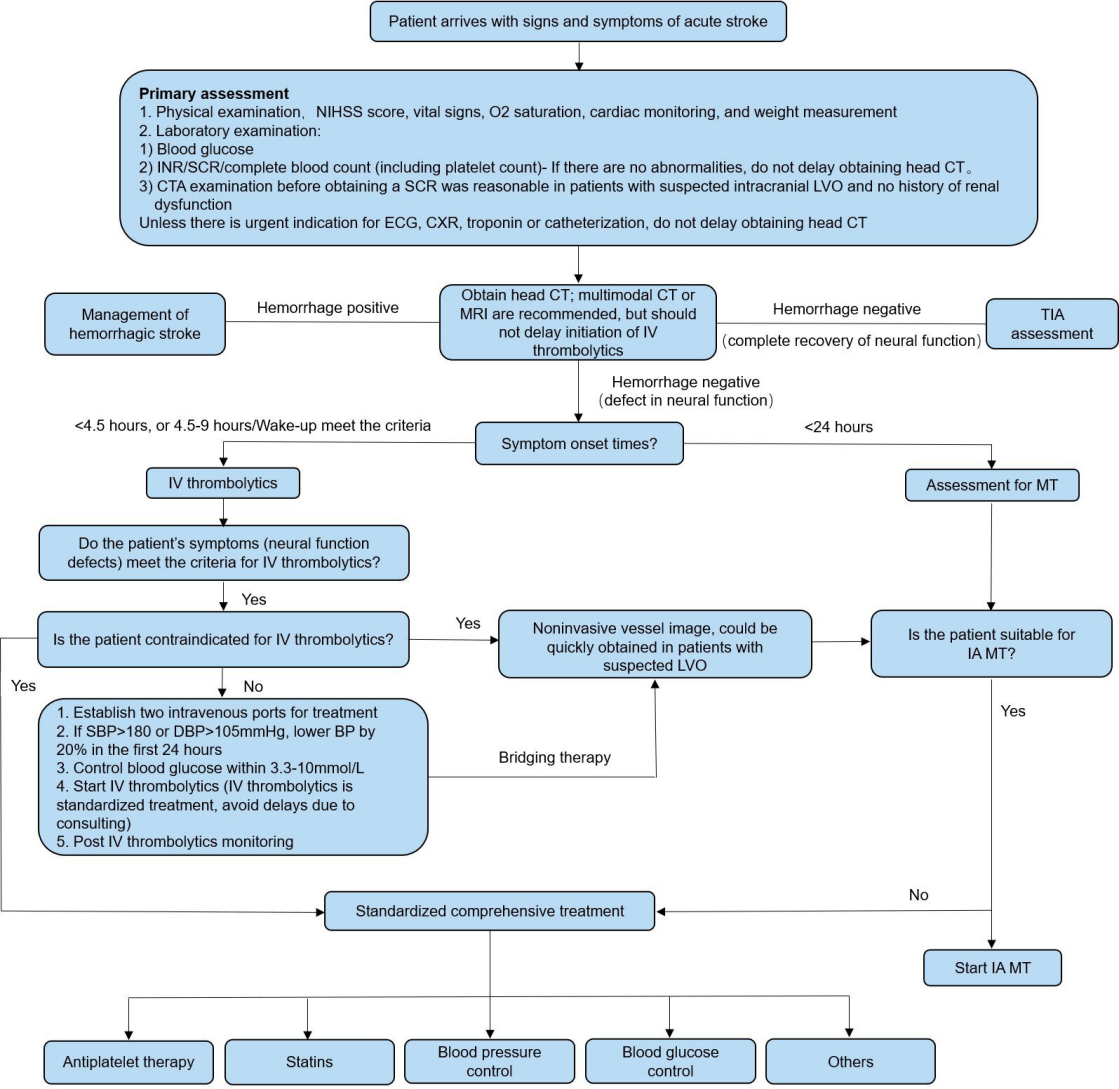
The acute care process for patients with acute ischaemic stroke. BP, blood pressure; CTA, CT angiography; CXR, chest X-ray; DBP, diastolic BP; IA, intra-arterial; INR, international normalised ratio; IV, intravenous; LVO, large vessel occlusion; MT, mechanical thrombectomy; NIHSS, National Institutes of Health Stroke Scale; SBP, systolic BP; SCR, serum creatinine; TIA, transient ischaemic attack.

**Figure 2:**
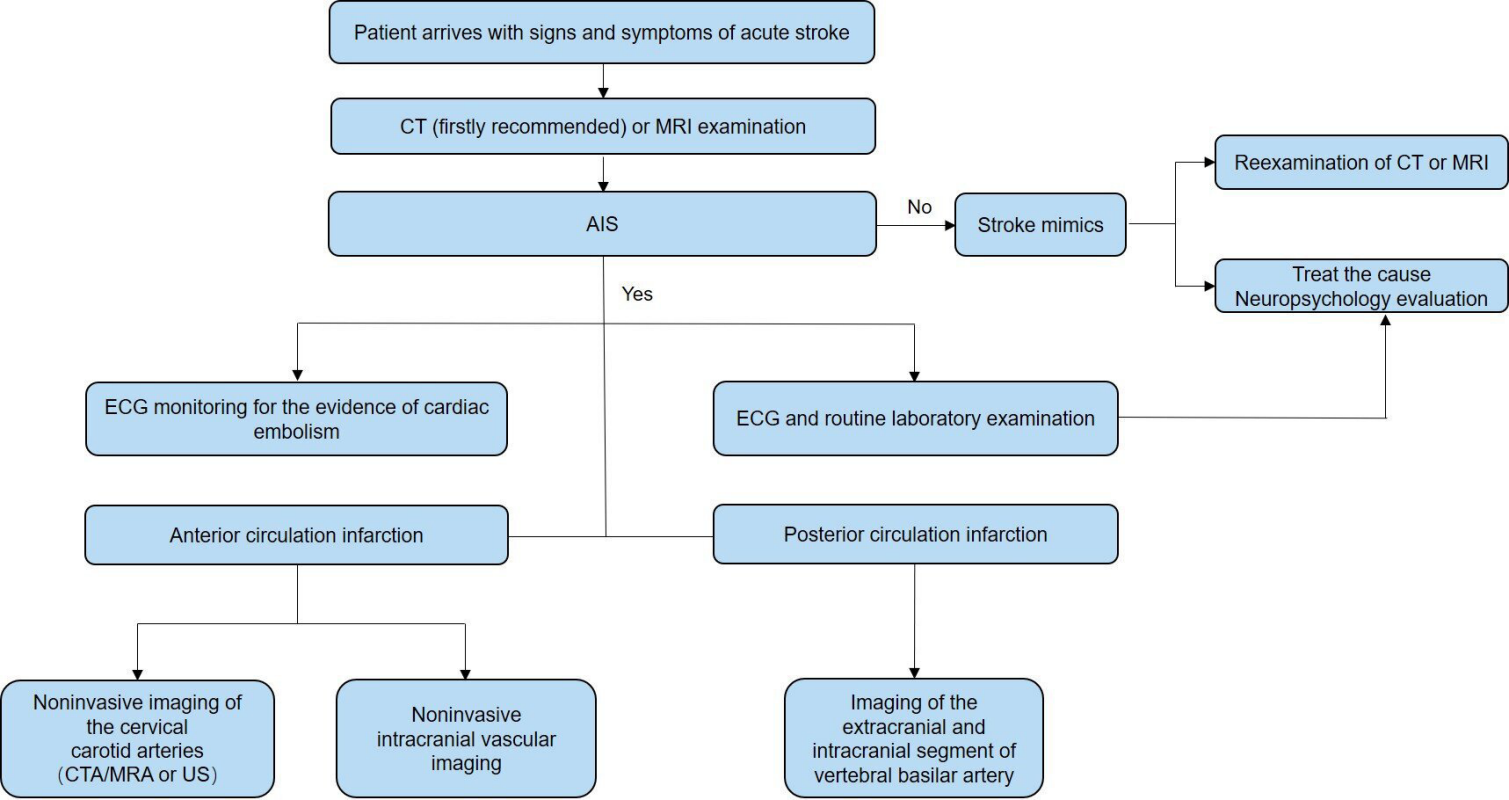
The emergency diagnostic and examination process flow chart for patients with acute ischaemic stroke (AIS). CTA, CT angiography; MRA, magnetic resonance angiography; US, ultrasonography.

The acute care process for patients with AIS is shown in figure 1. The emergency diagnostic and examination process flow chart for patients with AIS is shown in figure 2.

**Table:** 

Section 2: emergency assessment and diagnosis		Class of recommendation (COR)	Level of evidence (LOE)
Reworded	An AIS assessment team, consisting of physicians and nurses, should be established to conduct meticulous and standardised neurological examinations.	I	B
	Trained stroke emergency providers can rapidly and accurately identify a stroke and safely treat patients who had a stroke with intravenous rt-PA or TNK, and/or IA MT.^7^		
Reworded	The NIHSS score is recommended to assess stroke severity.	I	B
New recommendation	Dedicated image systems should be established to provide early neuroimaging examinations for patients who may qualify for intravenous thrombolysis and/or IA MT.	I	B
	The earlier patients complete the neuroimaging examinations, the sooner they can receive intravenous thrombolysis or IA MT, thus increasing the likelihood of re-establishing the perfusion.^8–12^		
New recommendation	Emergency brain image assessment should be conducted in all patients on the first arrival with suspected acute stroke before receiving any specific therapy.	I	A
	Brain imaging helps physicians diagnose intracranial haemorrhage (ICH), assess for infarction size, location, vascular distribution, severity, and find an LVO, and make immediate and long-term treatment decisions.^13–16^ For some patients with stroke upon wake-up, or unknown onset time, or within 4.5–9 hours, multimode brain imaging helps identify patients with AIS who may benefit from intravenous thrombolysis or IA MT and guide further treatment plans.^17–19^		
Reworded	Patients with suspected AIS should preferably undergo brain imaging within 30 min upon arrival at the emergency department.	I	B
New recommendation	A non-contrast CT (NCCT) scan is the first to be done to rule out an ICH. Then, initiate thrombolytic therapy as soon as possible.	I	B
	Although MRI and NCCT have equal efficiency in excluding ICH, NCCT is faster to identify an ICH (MRI: 13 min (10–16); NCCT: 9 min (7–12); p<0.001).^20–23^ Patients who had MRI and were treated with intravenous rt-PA or IA MT had a significant intrahospital delay of about 20 min.^24^ As the benefit of intravenous thrombolysis is time independent, NCCT should be completed as quickly as possible. Treatment should not be delayed by considering multimodal MRI or CT imaging.		
Reworded	In patients qualified for thrombolysis, initiating thrombolytic therapy should not be delayed by considering multimodal CT or magnetic resonance perfusion (MRP) studies.	I	B
New recommendation	For patients with wake-up stroke, stroke with an unknown onset time, or stroke occurring within 6–24 hours, CT angiography (CTA)+CT perfusion (CTP) or magnetic resonance angiography (MRA)+MRI is recommended to assess the potential benefits of intravenous thrombolysis or IA MT.	IIa	A
	In the DAWN trial, the NIHSS scores and core infarction mismatch on CTP or diffusion-weighted imaging (DWI) are used as eligible criteria for selecting patients with anterior circulation LVO within 6–24 hours for IA MT. The 90-day functional outcome in the MT group was significantly superior to that in the control group (modified Rankin Scale (mRS) 0–2, 49% vs 13%, adjusted difference 0.33, 95% CI 21 to 44).^18^ The DEFUSE 3 trial used CTP or DWI–perfusion-weighted imaging (PWI) perfusion-core mismatch and maximum core size to select patients with anterior circulation LVO within 6–16 hours for IA MT. It terminated early since patients treated with IA MT did better than those who received standard medical therapy (mRS 0–2, 44.6% vs 16.7%, rate ratio (RR)=2.67, 95% CI 1.60 to 4.48, p<0.001).^25^ The MR WITNESS trial used a quantitative mismatch of DWI-MRI with fluid-attenuated inversion recovery (FLAIR) to select patients for intravenous rt-PA with an onset >4.5 hours. About 39% of patients achieved mRS 0–1 at 90 days. One patient had an sICH (1.3%), and three developed symptomatic oedema (3.8%).^19^		
Reworded	Patients with suspected LVO and without a history of renal impairment can have the head and neck CTA first before obtaining serum creatinine to avoid delay in treatment.	I	B
New recommendation	For patients with AIS upon wake-up or unknown time of onset >4.5 hours, if MRI showed a DWI-positive/FLAIR-negative region of infarct, intravenous thrombolysis can be considered.	IIa	B
	The WAKE-UP trial selected patients who had a stroke with an unclear onset time >4.5 hours and an ischaemic lesion visible on DWI but no parenchymal hyperintensity on FLAIR for intravenous rt-PA. These patients had a significantly better functional outcome (53.3% vs 41.8%, adjusted OR 1.61, 95% CI 1.09 to 2.36, p=0.02) but also more sICH (2.0% vs 0.4%, adjusted OR 4.95, 95% CI 0.57 to 42.87, p=0.15) than placebo at 90 days.^17^ For patients with AIS upon wake-up, or within 4.5–9 hours, the benefits of intravenous rt-PA in patients with AIS with an imaging mismatch were associated with better outcomes and showed no significant differences in the risk of ICH.^9^		
New recommendation	For patients with suspected LVO, MRA or CTA should be completed as soon as possible to determine the eligibility for IA MT.	I	A
	Identification of an LVO requires either a CTA or MRA. Two comparative studies evaluated the sensitivity of CTA, MRA and digital subtraction angiography (DSA) to diagnose intracranial stenosis and occlusion.^26 27^ CTA had a significantly higher positive predictive value for stenosis (93% vs 65%, p<0.001) and occlusion (100% vs 59%, p<0.001) than MRA.^26 27^ The sensitivity of CTA and MRA for the diagnosis of LVO ranges from 87% to 100% compared with the gold-standard DSA.^27^ As the efficacy of IA MT is time-dependent, the vascular image should be conducted as quickly as possible.^28^		
New recommendation	For patients with indications for IA MT, performing a vascular imaging of extracranial carotid and vertebral arteries helps the approach for IA MT.	IIb	C
	Most studies focused on the effectiveness of IA MT for AIS excluded patients with tandem occlusions. A retrospective review showed that treating tandem extracranial carotid artery steno-occlusion with IA MT, 42% had better outcomes and 88% had successful reperfusion.^29^		
New recommendation	For patients with indications for IA MT, the assessment of collateral flow may help select treatment.	IIb	C
	The MR CLEAN-LATE trial enrolled patients with collateral flow in the middle cerebral artery (MCA) territory of the affected hemisphere on CTA within 6–24 hours of onset and found IA MT was effective and safe.^30^ In the DAWN trial, better collaterals, defined with the Tan scale by CTA or the American Society of Interventional and Therapeutic Neuroradiology grade by DSA, were associated with slower stroke progression and better functional outcomes.^31^ Hypoperfusion intensity ratio (HIR), defined as Tmax >10 s volume/Tmax >6 s volume, was independently associated with the collateral status. Poor collateral status (high HIR) was related to rapid infarct growth in the DEFUSE trial.^32^		
Reworded	For patients with AIS with LVO in the anterior circulation presenting 6–24 hours after onset, it is recommended that CTP or DWI with PWI be completed. Patients selected for IA MT should follow the same eligibility criteria of the two major RCTs (DAWN and DEFUSE 3).	IIa	B
New recommendation	For patients with AIS with suspected BAO presenting between 6 and 24 hours of onset, CTA, MRA or DSA should be completed. Patients selected for BAO MT should follow the same eligibility criteria of the ATTENTION or BAOCHE.	IIa	B
	The ATTENTION trial used CTA/MRA/DSA to select patients with BAO within 6–12 hours of onset and a baseline NIHSS ≥10 for IA MT. About 46% of the patients treated with IA MT had better outcomes compared with 23% treated with the best medical care (adjusted RR, 2.06; 95% CI 1.46 to 2.91; p<0.001).^4^ The BAOCHE trial also used CTA/MRA/DSA to select patients with BAO within 6–24 hours of onset and a baseline NIHSS ≥10 for MT. About 46% treated with IA MT had better outcomes compared with 24% treated with the medical care (adjusted RR, 1.81; 95% CI 1.26 to 2.60; p<0.001). Both trials observed a higher rate of sICH in the IA MT group.^5^		
New recommendation	MRI is not routinely recommended to exclude cerebral microbleeds (CMBs) in patients eligible for intravenous thrombolysis.	I	A
	Although the presence of CMBs and high CMB burdens is related to sICH in patients treated with intravenous rt-PA, one meta-analysis indicated the prevalence of CMBs on pretreatment MRI was not associated with a higher risk of early sICH (OR 1.74, 95% CI 0.91 to 3.33, I^2^=44.5%).^33–36^ Besides, both NINDS and ECASS III Studies did not exclude these patients.^37 38^		
Unchanged	Less than 10 CMBs on MRI may be safe for intravenous thrombolysis.	IIa	B
Reworded	There is an increased risk of sICH in patients with >10 CMBs on pre-thrombolysis MRI. The clinical benefit of thrombolysis is unclear. If there may be significant potential benefits, intravenous thrombolysis may be reasonable.	IIb	B
Reworded	All patients should undergo blood glucose testing before intravenous thrombolysis.	I	B
Reworded	A baseline 12-lead ECG is recommended but should not delay the initiation of intravenous thrombolysis.	I	B
Reworded	Laboratory tests, including electrolytes, renal function, complete blood count with platelet count, coagulation function with international normalised ratio (INR) and cardiac ischaemia biomarkers, are recommended but should not delay the initiation of intravenous thrombolysis or IA MT.	I	C
Unchanged	Considering the low incidence of platelet abnormalities and coagulation dysfunction in the general population, intravenous thrombolysis should not be delayed while waiting for the results of platelet counts.	IIa	B
Revised	The benefits of chest X-rays in patients with hyperacute stroke are uncertain in the absence of evidence of acute pulmonary, cardiac or pulmonary vascular disease. If needed, it should not delay the initiation of intravenous thrombolysis.	IIb	B
	Chest radiographs could provide information on pulmonary disease, cardiac disease, cardioembolic stroke and even aortic dissection based on the mediastinal width-to-chest ratio on chest X-ray.^39 40^ However, patients with a chest X-ray had significantly higher door-to-needle time than those had it after the treatment (75.8 vs 58.3 min, p=0.0001).		
Reworded	The Alberta Stroke Program Early CT Score (ASPECTS) is recommended to provide guidance for IA MT. However, the decision-making doctor must have the training in calculating ASPECTS.	IIa	B
New recommendation	When assessing the benefits of IA MT for patients with AIS within 6 hours who have LVO in the anterior circulation and an ASPECTS ≥6, CT+CTA or MRI+MRA is preferably recommended.	I	A
	IA MT for patients with an ASPECTS ≥6 and an anterior circulation LVO is more effective than those with an ASPECTS <6.^41^ In the MR CLEAN, ESCAPE, EXTEND-IA, SWIFT PRIME, REVASCAT and THRACE trials, only THRACE and MR CLEAN trials used NCCT to select patients for IA MT, while the other four RCTs used CT+CTA or MRI+MRA.^42–47^		

### Section 3: reperfusion therapy for AIS

**Figure 3:**
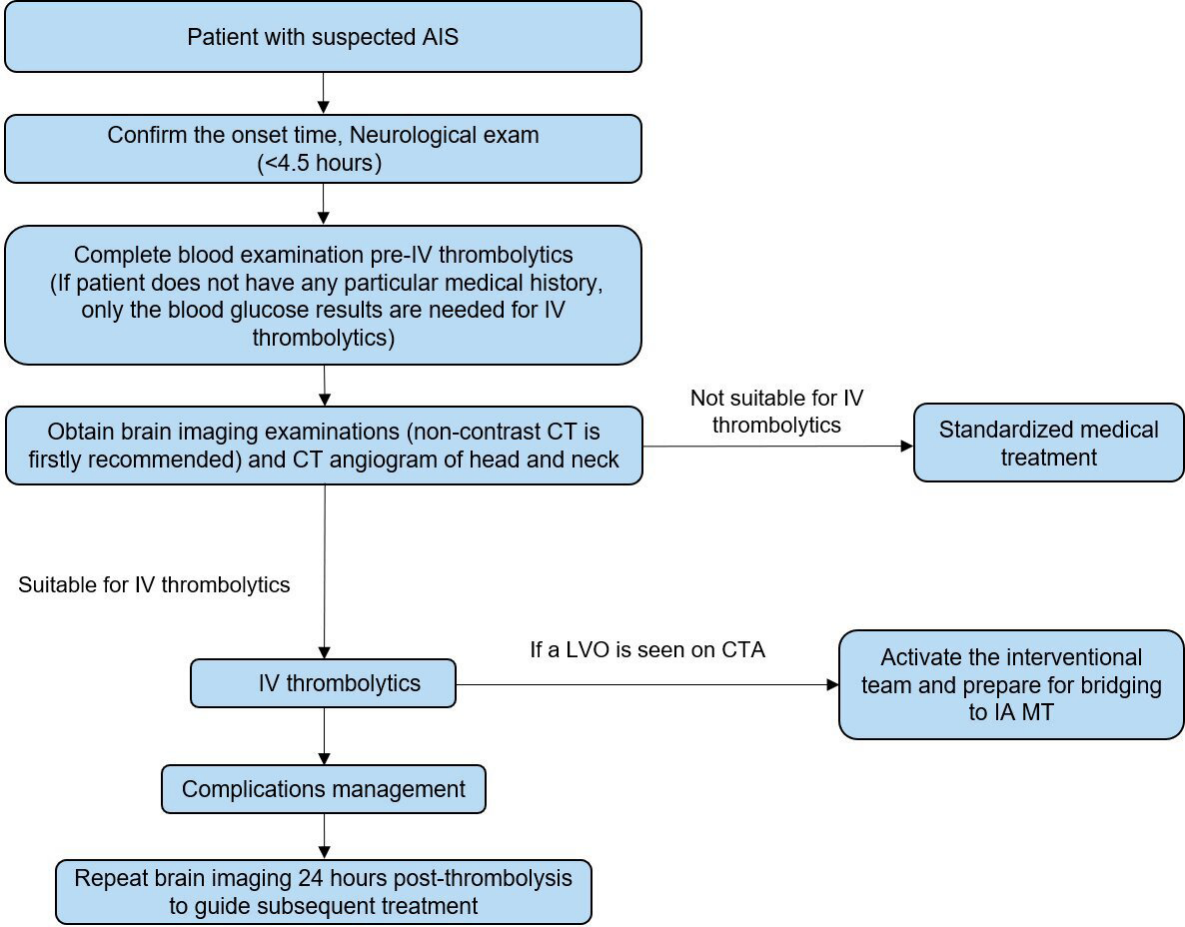
The management process of intravenous (IV) thrombolysis for patients with acute ischaemic stroke (AIS) within 4.5 hours of symptom onset. CTA, CT angiography; IA, intra-arterial; LVO, large vessel occlusion; MT, mechanical thrombectomy.

**Figure 4:**
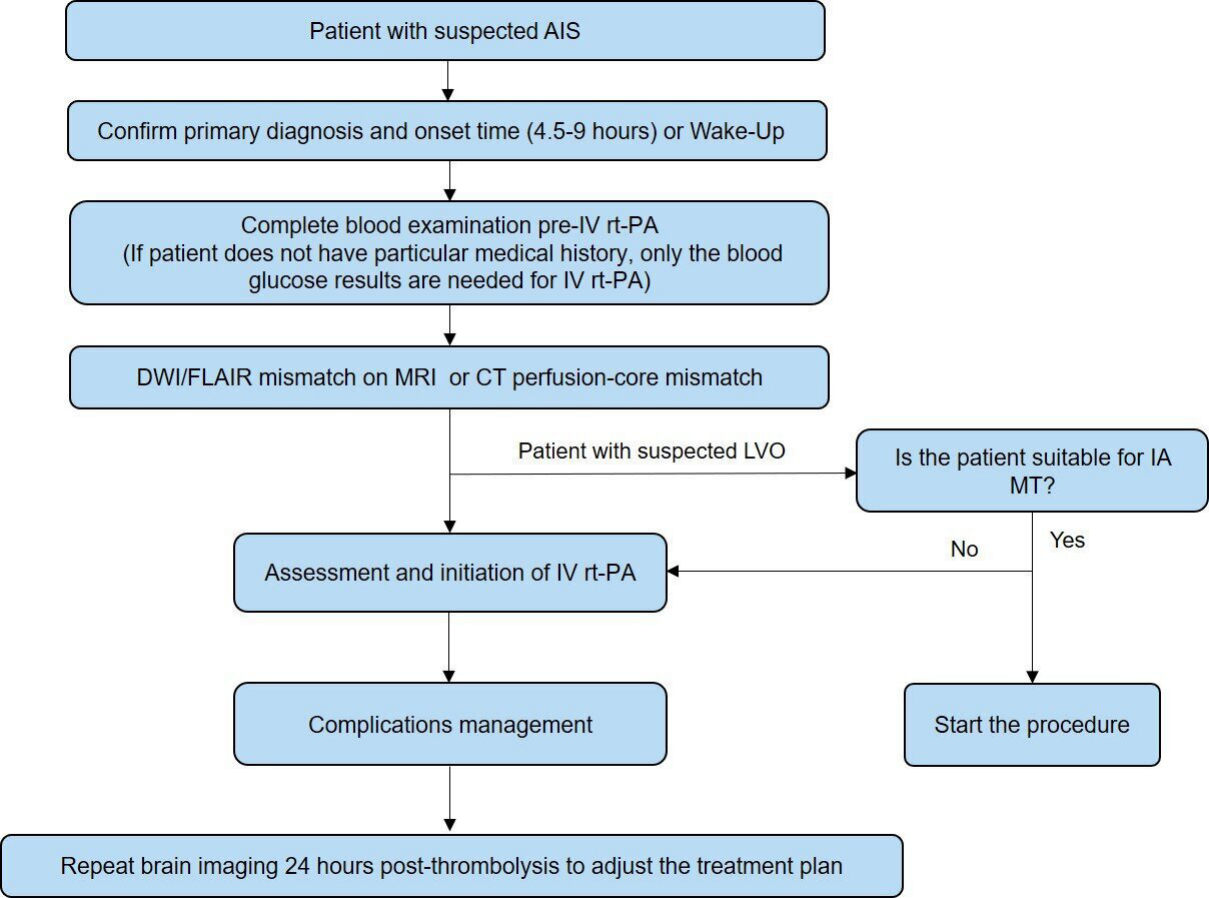
The management process of intravenous (IV) thrombolysis for patients with acute ischaemic stroke (AIS) with an onset between 4.5 and 9 hours or wake-up stroke. DWI, diffusion-weighted imaging; FLAIR, fluid-attenuated inversion recovery; IA, intra-arterial; LVO, large vessel occlusion; MT, mechanical thrombectomy; rt-PA, recombinant tissue plasminogen activator.

**Figure 5:**
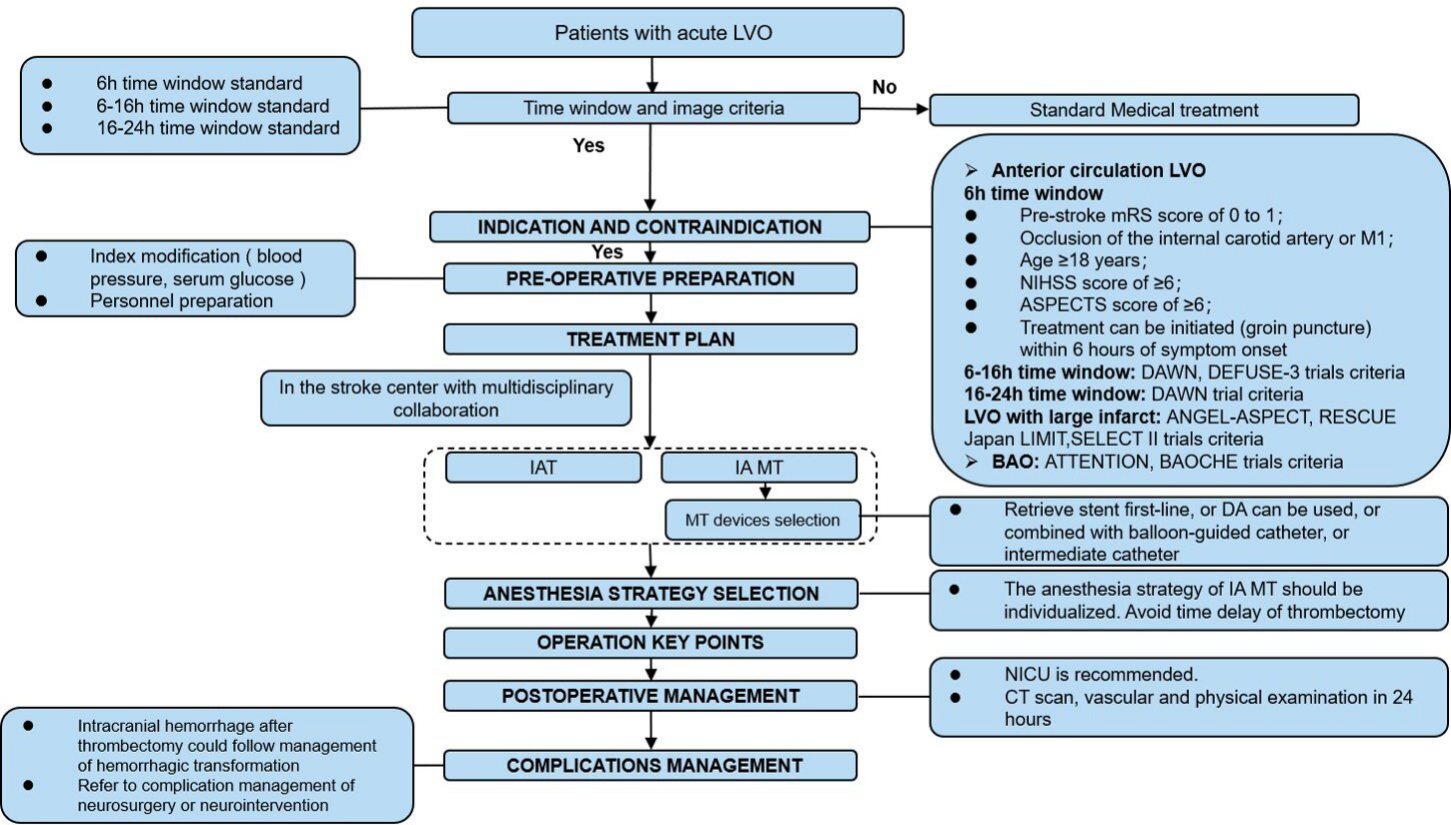
The intra-arterial (IA) mechanical thrombectomy (MT) for patients with acute ischaemic stroke. ASPECTS, Alberta Stroke Program Early CT Score; BAO, basilar artery occlusion; DA, direct aspiration; IAT, intra-arterial thrombolysis; IV, intravenous; LVO, large vessel occlusion; mRS, modified Rankin Scale; NICU, neurological intensive care unit; NIHSS, National Institutes of Health Stroke Scale.

The management process of intravenous thrombolysis for patients with AIS within 4.5 hours of symptom onset is shown in figure 3. The management process of intravenous thrombolysis for patients with AIS with an onset between 4.5 and 9 hours or wake-up stroke is shown in figure 4. The flow chart for IA MT in patients with AIS is shown in figure 5.

#### Section 3.1 Intravenous thrombolysis

**Table:** 

Table 3.1 Intravenous thrombolysis		COR	LOE
Revised	For patients suitable for intravenous thrombolysis within 4.5 hours of onset, intravenous rt-PA treatment is recommended (0.9 mg/kg, maximum dose 90 mg, 10% of the total dose intravenous push over 1 min, and the rest intravenous drip within 60 min).	I	A
	The ENCHANTED trial did not show non-inferiority of the low dose of rt-PA (0.6 mg/kg) in functional outcome recovery compared with the standard dose.^48^ A dose of 0.9 mg/kg intravenous rt-PA was used in most RCTs focused on the benefit of thrombolysis within 4.5 hours. For Chinese patients with AIS within 3–4.5 hours after symptom onset, the standard dose of rt-PA (0.9 mg/kg) for intravenous thrombolysis is recommended as effective and safe.^49^ The ECASS III trial found that intravenous rt-PA administered between 3 and 4.5 hours after the onset of symptoms significantly improved clinical outcomes in patients with AIS.^38^		
Revised	For elderly patients with AIS (aged >80 years) with an onset <4.5 hours, intravenous rt-PA treatment is considered reasonable.	IIa	B
	In clinical trials, intravenous rt-PA has been restricted to patients aged 18–80 years old. Systematic review revealed a similar benefit in patients >80 years, particularly when treated early.^50 51^ A higher proportion of mRS 0–1 (19.1% vs 13.1%, p=0.0109) and similar 90 days’ mortality (29.5% vs 30.2%, p=0.8382) were associated with intravenous rt-PA versus placebo.^50^		
New recommendation	For patients with AIS occurring beyond 4.5 hours but with DWI lesions, less than one-third of the MCA territory or no visible lesions on FLAIR, intravenous rt-PA is beneficial.	IIa	B
New recommendation	Intravenous rt-PA is not recommended for patients with AIS within 4.5–9 hours based only on a NCCT.	III	B
	In the WAKE-UP, MR WITNESS, EXTEND and EPITHET trials, patients with AIS within 4.5–9 hours of onset (including unknown time of onset and wake-up) had either CT+CTP or DWI+FLAIR for eligibility of intravenous rt-PA or not.^9 17 19^ Intravenous rt-PA-treated patients had improved outcomes.		
New recommendation	For patients with AIS within 4.5–9 hours (including unknown time of onset), if the perfusion-core mismatch identified by CTP or MRP indicates the benefits from vascular recanalisation treatment, intravenous rt-PA treatment is recommended when IA MT is not planned or recommended.	I	A
	The EXTEND and EPITHET trials showed a valuable use of multimodel CT or MRI to identify patients with AIS within 4.5–9 hours or unknown time of onset who would benefit from intravenous rt-PA.^52 53^		
New recommendation	For patients who had a wake-up stroke, if DWI–FLAIR mismatch indicates the benefits from vascular recanalisation treatment, intravenous rt-PA treatment is recommended when IA MT is not planned or recommended.	IIa	B
	For patients with wake-up AIS and treated based on the perfusion-core mismatch, both IA MT and intravenous rt-PA of an LVO improve functional outcomes without increasing the risk of death. The EXTEND trial used CTP or MRP, while the WAKE-UP trial used DWI–FLAIR mismatch to select patients for intravenous rt-PA.^17 52^		
Unchanged	Treat hypoglycaemia (blood glucose <60 mg/dL) in patients with AIS.	I	C
Unchanged	Prolonged hyperglycaemia within 24 hours of hospitalisation is associated with adverse outcomes compared with normal blood glucose levels. It is recommended correcting hyperglycaemia and maintaining it between 140 and 180 mg/dL while closely monitoring it to prevent hypoglycaemia.	IIa	C
Revised	Patients with elevated blood pressure but otherwise suitable for intravenous rt-PA treatment should be cautious in lowering blood pressure before thrombolysis. The recommended goal of systolic blood pressure (SBP) is <185 mm Hg and diastolic blood pressure (DBP) is <110 mm Hg.	I	B
	Increased blood pressure is associated with a higher risk of sICH after intravenous rt-PA treatment. The NINDS and ECASS trials excluded patients with baseline blood pressure >185/110 mm Hg.^37 54^ The IST-3 and ECASS III trials did not mention the restrictions on blood pressure.^38 55^ In an observational study, pretreatment blood pressure of SBP >185 mm Hg and/or DBP >110 mm Hg was independently associated with a higher risk of sICH (OR 2.59, 95% CI 1.07 to 6.25).^56^		
Revised	For patients who have not received intravenous thrombolysis but are planned for IA MT, it may be reasonable to maintain blood pressure ≤185/110 mm Hg before the procedure.	IIb	B
	The REVASCAT, SWIFT PRIME, EXTEND-IA, TRACE, MR CLEAN and DAWN trials excluded patients with baseline blood pressure >185/110 mm Hg for bridging treatment.^18 42 45–47 57^ It is reasonable to control preoperative blood pressure below 185/110 mm Hg before IA MT.		
Revised	During intravenous thrombolysis and within 24 hours after treatment, blood pressure should be maintained at <180/105 mm Hg.	I	B
	The ENCHANTED trial compared controlling blood pressure to either >150 mm Hg and <185/110 mm Hg, or to 130–140 mm Hg within 72 hours after intravenous rt-PA. No significant difference in mRS was found at 90 days.^58^ However, another RCT with 626 patients who underwent thrombolysis showed a significant decrease in the risk of ICH and mortality if SBP was kept between 141 and 150 mm Hg, compared with those kept SBP between 151 and 185 mm Hg.^59^		
New recommendation	The risk of using antithrombotic therapy within 24 hours after intravenous thrombolysis is still uncertain.	IIb	B
	A prospective clinical registry study revealed that earlier initiation of antithrombotics was associated with a low risk of any haemorrhage (adjusted OR 0.56, 95% CI 0.35 to 0.89), or sICH (adjusted OR 0.85, 95% CI 0.35 to 2.10) or mRS 0–3 changes at 90 days (adjusted OR 1.09, 95% CI 0.75 to 1.59).^60^ In the post hoc analysis of the randomised ARTIS trial, early aspirin use after intravenous rt-PA increased the risk of sICH (OR 3.73, 95% CI 1.03 to 13.49) but not cerebral ischaemia (OR 1.14, 95% CI 0.44 to 3.00).^61^		
New recommendation	Intravenous administration of aspirin should not be used within the first 90 min of initiating intravenous thrombolysis.	III	B
	The ARTIS trial randomly assigned patients to 300 mg intravenous aspirin within 90 min after starting rt-PA therapy or to no additional treatment. Oral antiplatelet therapy was started 24 hours after intravenous rt-PA treatment. This trial was stopped early due to an excess of sICHs (4.3% vs 1.6%, absolute difference 2.8%, 95% CI 0.2% to 5.4%, p=0.04) and no benefit in outcome (54.0% vs 57.2%, absolute difference −3.2%, 95% CI −10.8% to 4.2%, crude relative risk 0.94, 95% CI 0.82 to 1.09, p=0.42) in the aspirin group.^62^		
New recommendation	Patients with AIS occurring within <4.5 hours and presenting with multiple comorbidities, weakness or pre-stroke disabilities may also benefit from intravenous thrombolysis.	IIb	B
	For patients with chronic kidney disease (CKD) or renal dysfunction and treated with intravenous rt-PA, two meta-analyses demonstrated a higher risk of sICH and poor functional outcome, mainly in patients with moderate-to-severe CKD.^63 64^ Another meta-analysis showed a neutral result.^65^ Patients diagnosed with cancer may also benefit from intravenous thrombolysis without increased risk of bleeding.^66^ Patients with pre-existing disabilities before intravenous rt-PA treatment, those with mRS scores 2 and ≥3 had similar favourable functional outcomes (34% vs 29%), despite higher mortality (48% vs 39%).^67^ Patients with pre-existing disabilities had a higher risk of mortality (33% vs 14%, OR 3.2, 95% CI 1.0 to 10.1) and poor function outcome (median mRS 3 vs 2, p=0.03), but little difference in a good NIHSS score at 24 hours or 3 months, and favourable outcomes at 3 months.^68^		
New recommendation	For patients with AIS with mild and disabling symptoms within 4.5 hours of onset, intravenous thrombolysis is recommended.	IIa	B
	Mild or rapidly improving strokes were not well studied in clinical trials. A post hoc analysis of the NINDS and IST-3 trials showed the benefit of rt-PA in patients with minor stroke compared with the control group.^69 70^ In the MINOR-STROKE trial, patients with M1 LVO and minor stroke symptoms benefited from bridging therapy, whereas patients with M2 LVO benefited from intravenous rt-PA alone.^71^ Data from the Austrian Stroke Unite Registry divided 35 113 patients into NIHSS 0–1 for non-disabling stroke and NIHSS 2–4 for mild deficit stroke.^72^ Intravenous rt-PA in NIHSS 0–1 group was associated with an early neurological deterioration (adjusted OR 8.84, 95% CI 6.61 to 11.83), increased risk of sICH (adjusted OR 9.32, 95% CI 4.53 to 19.15) and a lower rate of excellent outcome (mRS 0–1) at 90 days, whereas patients with NIHSS 2–5 had a higher rate of excellent outcomes (mRS 0–1), but initially neurological deterioration (adjusted OR 1.7, 95% CI 1.47 to 1.98) and sICH (adjusted OR 5.75, 95% CI 4.45 to 7.45).		
New recommendation	For patients with AIS with mild non-disabling symptoms (NIHSS 0–5) within 4.5 hours, intravenous thrombolysis is not routinely recommended.	III	B
	The PRISMS trial enrolled 313 patients who had minor non-disabling neurological deficits with an NIHSS score of 0–5. Favourable outcome was seen in 78.2% of patients in the intravenous rt-PA group vs 81.5% in the aspirin group (adjusted risk difference −1.1%, 95% CI −9.4% to 7.3%). About 3.2% of patients in the intravenous rt-PA group had sICH, but none in the aspirin group (risk difference 3.3%, 95% CI 0.8% to 7.4%).^73^ More evidence is needed, and the MaRISS trial is ongoing.^74^		
Reworded	Intravenous thrombolysis is not suitable for patients who have used low molecular weight heparin (LMWH) within 24 hours, regardless of whether the dose is for prophylactic or therapeutic purposes.	III	B
Unchanged	During intravenous thrombolysis, physicians should be fully prepared to respond to adverse reactions such as haemorrhagic complications and vasogenic oedema.	I	B
Unchanged	The safety and efficacy of intravenous thrombolysis in patients with AIS with coagulation disorders have not been determined.	III	C
Reworded	Any delay in intravenous thrombolysis has a major impact on prognosis. The treatment should not be postponed to wait for symptoms to resolve spontaneously.	III	C
Reworded	Intravenous thrombolysis may benefit patients with AIS with a digestive tract or urinary bleeding history.	IIb	C
Reworded	Intravenous thrombolysis for patients with AIS may be considered within 14 days of surgery. However, careful consideration is required regarding the risk of surgical site bleeding and the benefits of thrombolysis.	IIb	C
Reworded	In patients with AIS with a history of major trauma (within 14 days, without affecting the head), intravenous thrombolysis can be carefully considered. The risk of wound haemorrhage versus the severity of stroke and subsequent disability should be taken into consideration.	IIb	C
Reworded	The safety and efficacy of intravenous thrombolysis in patients with AIS with a history of vascular perforation within 7 days are still uncertain.	IIb	C
Reworded	For patients with AIS with lumbar punctures within 7 days, the safety of intravenous thrombolysis is uncertain.	IIb	C
Unchanged	In patients with AIS with abnormal baseline glucose (<50 mg/dL (2.78 mmol/L) or >400 mg/dL (22.2 mmol/L)), the benefit of intravenous thrombolysis is uncertain once the abnormal glucose level is corrected.	IIb	C
Reworded	In patients with AIS presenting with seizures, if evidence suggests that limb dysfunction is due to stroke rather than post-seizure paralysis, intravenous thrombolysis may be beneficial.	IIa	C
Reworded	In patients with AIS with a known or suspected extracranial carotid artery dissection and stroke symptom onset <4.5 hours, intravenous thrombolysis should be cautiously considered.	IIa	C
Reworded	The efficacy and safety of intravenous thrombolysis have not been established in patients with AIS with a known or suspected intracranial carotid artery dissection.	IIb	C
Reworded	In patients with AIS with a small or moderate-sized (<10 mm) unruptured intracranial aneurysm, intravenous thrombolysis may be cautiously considered.	IIa	C
Reworded	In patients with AIS with a large unruptured or unstable intracranial aneurysm, the efficacy and safety of intravenous thrombolysis are uncertain.	IIb	C
Reworded	In patients with AIS with unruptured or untreated intracranial vascular malformations, the efficacy and safety of intravenous thrombolysis are not known.	IIb	C
Reworded	Patients with AIS with neuroectodermal tumours may benefit from intravenous thrombolysis.	IIa	C
Reworded	In patients with AIS with an acute myocardial infarction (MI), consideration can be given to administering intravenous thrombolysis with an appropriate dose for AIS, followed by percutaneous coronary intervention or stent placement for acute coronary syndrome.	IIa	C
Reworded	In patients with AIS with a recent MI (>3 months), intravenous thrombolysis may be beneficial if it is a non-ST-elevation MI, or an ST-elevation MI involving the right ventricle/inferior wall.	IIa	C
Reworded	In patients with AIS with a recent MI (>3 months), the safety and risk of intravenous thrombolysis are uncertain if ST is elevated and involves the left ventricle/anterior wall.	IIb	C
Unchanged	In severe AIS with acute pericarditis, which may lead to severe disability (mRS 3–5), the benefit of intravenous thrombolysis is not clear. An urgent cardiologist consultation is required.	IIb	C
Unchanged	In patients with mild or moderate AIS with acute pericarditis or left atrial/ventricular thrombus, the risk and benefit of intravenous thrombolysis are unknown.	III	C
Unchanged	Severe AIS related to a left atrial/ventricular thrombus, atrial myxoma or papillary fibroids may have severe disability (mRS 3–5). The safety and efficacy of intravenous thrombolysis are unknown.	IIb	C
Reworded	After patients with AIS undergo cardiovascular or cerebrovascular DSA, intravenous thrombolysis may be beneficial. Patients should be carefully assessed for indications, contraindications and relative contraindications.	IIa	A
Reworded	The efficacy and safety of intravenous thrombolysis in patients with AIS with malignancy are unknown. If the expected survival period is >6 months, with no other contraindications or coagulopathy or bleeding, careful consideration of intravenous thrombolysis can be considered.	IIb	C
Unchanged	Pregnant women with moderate or severe stroke may benefit from intravenous thrombolysis if the benefits outweigh the risk of intrauterine bleeding.	IIb	C
Unchanged	The benefits and risks of intravenous thrombolysis for patients with AIS within 14 days of post partum are non-conclusive.	IIb	C
Reworded	Urokinase is safe for those unsuitable for intravenous rt-PA treatment within 6 hours of onset. However, the validity needs further confirmation by high-quality RCTs with large sample size.	IIb	B
	In a multicentre retrospective study, urokinase had similar functional independence in Chinese patients with AIS, but with a higher risk of extracranial bleeding compared with patients treated with rt-PA.^75^		
New recommendation	TNK 0.4 mg/kg is harmful to treat patients with AIS with an NIHSS score >6.	III	B
	The NOR-TEST2 Part A trial involved patients with NIHSS scores ≥ 6 within 4.5 hours. Patients who accepted TNK (0.4 mg/kg) had a lower likelihood of favourable functional outcome (32% vs 51%, OR 0.45, 95% CI 0.25 to 0.80, p=0.0064), more frequent ICH (21% vs 7%, OR 3.68, 95% CI 1.49 to 9.11, p=0.0031) and a higher rate of mortality at 3 months (16% vs 5%, OR 3.56, 95% CI 1.24 to 10.21, p=0.013).^76^		
New recommendation	TNK 0.25 mg/kg intravenous push has been proven non-inferior to intravenous standard dosage of rt-PA to treat patients with AIS with <4.5 hours of onset.	IIa	B
	In the TASTE-A study, TNK 0.25 mg/kg (maximum 25 mg) was associated with a smaller perfusion lesion volume (median 12 mL vs 35 mL, adjusted OR 0.55, 95% CI 0.37 to 0.81, p=0.0030) compared with rt-PA 0.9 mg/kg (maximum 90 mg) in patients with AIS treated within 4.5 hours, with the non-significant difference in safety outcomes.^77^ The AcT trial indicated a non-inferiority of TNK (0.25 mg/kg) in the functional outcome compared with rt-PA (36.9% vs 34.8%, unadjusted risk difference 2.1%, 95% CI 2.6% to 6.9%) in patients with AIS treated within 4.5 hours.^78^ The TRACE-2 trial found that, compared with rt-PA 0.9 mg/kg (maximum 90 mg), recombinant human TNK 0.25 mg/kg (maximum 25 mg) showed an efficacy (62% vs 58%, RR 1.07, 95% CI 0.98 to 1.16), which was non-inferior to rt-PA in patients eligible for intravenous thrombolysis with AIS.^79^		
New recommendation	For patients with anterior circulation LVO type of AIS who present within 4.5 hours of symptom onset, the efficacy of intravenous TNK (0.25 mg/kg) is non-inferior to intravenous rt-PA (0.9 mg/kg) before IA MT. The TNK might offer better reperfusion outcomes, while the incidence of sICH remains similar.	IIa	B
	The EXTEND-IA TNK trial focusing on the efficacy and safety of TNK (0.25 mg/kg) and rt-PA (0.9 mg/kg) before thrombectomy revealed that 22% of patients in the TNK group were reperfused >50% of the affected vascular area compared with 10% in the rt-PA group.^80^ The rate of any ICH was 3% in the TNK group and 2% in the rt-PA group. Patients in the TNK group also showed a better functional outcome (mRS 2 vs 3) at 90 days than those treated with rt-PA. In the TEMPO-1 trial, 66% had excellent functional outcomes at 90 days and a high recanalisation rate: 0.1 mg/kg (39% complete and 17% partial), 0.25 mg/kg (52% complete and 9% partial).^81^ Administration of TNK in patients with minor stroke and anterior circulation LVO is feasible and safe.		
Reworded	Besides performing in clinical trials, ultrasound-assisted thrombolysis is not recommended as an adjunct treatment to intravenous thrombolysis. Similarly, image-guided desmoteplase thrombolysis is not recommended.	III	B
New recommendation	Other fibrinolytic agents and thrombolytics, except for rt-PA, TNK and urokinase, are not recommended.	III	B

#### Section 3.2 Bridging/MT

**Table:** 

Table 3.2 Bridging/MT		COR	LOE
Reworded	IA MT is strongly recommended for patients with AIS within 6 hours of onset and meet the following criteria: (1) pre-stroke mRS score of 0–1; (2) AIS caused by occlusion of the distal internal carotid artery (ICA) or MCA M1 segment; (3) age ≥18 years old; (4) NIHSS score ≥6; (5) ASPECTS ≥6.	I	A
New recommendation	For patients with AIS with anterior circulation LVO and a large core infarct within 24 hours of onset and who meet the inclusion criteria of the RESCUE-Japan LIMIT, ANGEL-ASPECT and SELECT 2 trials, IA MT is recommended.	I	A
	Three RCTs (RESCUE-Japan LIMIT, ANGEL-ASPECT and SELECT 2) have demonstrated that IA MT is superior to medical management (MM) in treating patients with AIS with a large core infarction from an anterior circulation LVO.^82–84^ However, the imaging modality of the baseline large infarcts was different among the three trials. The RESCUE-Japan LIMIT trial enrolled patients who had ICA or M1 occlusions with an ASPECTS between 3 and 5 by DWI or NCCT (most DWI) and within 6 hours from onset to randomisation or no signal change in the initial image on FLAIR with ASPECTS 3–5 within 6–24 hours from the onset to randomisation.^82^ The ANGEL-ASPECT trial enrolled patients with AIS within 24 hours of onset with an ASPECTS between 3 and 5 on NCCT or 0–2 on NCCT with infarct-core volume between 70 and 100 mL.^83^ The SELECT 2 trial enrolled patients with a large infarct and the ASPECTS between 3 and 5 on NCCT or infarct-core volume >50 mL.^84^		
Reworded	For patients with occlusion in the anterior cerebral artery or posterior cerebral artery, IA MT may be considered within 6 hours of symptom onset.	IIb	C
Reworded	For patients with occlusion in the cervical ICA or MCA M1 segment, and a pre-stroke mRS score >1, or NIHSS score <6, the use of stent-retriever IA MT may be considered within 6 hours of symptom onset (time of femoral artery puncture).	IIb	B
Reworded	For patients with AIS with an anterior circulation LVO within 6–16 hours of last known normal, IA MT is strongly recommended when they meet the inclusion criteria of the DAWN or the DEFUSE 3 study.	I	A
Reworded	For patients with AIS with an anterior circulation LVO within 16–24 hours of the last known normal, IA MT is recommended when they meet the inclusion criteria of the DAWN study.	IIa	B
New recommendation	For patients with acute BAO within 6 hours of onset who meet the inclusion criteria of the ATTENTION trial, IA MT is recommended.	IIb	B
	Patients with acute BAO within 6–12 hours of onset are recommended for IA MT when they meet the inclusion criteria of the ATTENTION or BAOCHE trials.	IIa	A
	Patients with acute BAO within 12–24 hours of onset are recommended for MT when they meet the inclusion criteria of the BAOCHE trial.	IIa	B
	Among the BEST, BASICS, ATTENTION and BAOCHE trials that treated BAO,^4 5 85 86^ the BEST (42% vs 32%) and BASICS (44.2% vs 37.7%) trials did not find the superiority of IA MT to MM in patients with acute BAO within 8 hours or 6 hours of onset.^85 86^ The ATTENTION (46% vs 23%) and BAOCHE (46% vs 24%) trials found that IA MT is superior to MM in patients with acute BAO within 12 hours or 6–24 hours of onset.^4 5^		
New recommendation	During IA MT, IA thrombolysis with rt-PA at a dose of 0.225 mg/kg can be performed in patients with a modified Thrombolysis in Cerebral Infarction 2b50 recanalisation even though these patients may have received intravenous thrombolysis before IA MT.	IIa	B
	The CHOICE study explored whether adjuvant IA rt-PA can improve the clinical outcomes of patients after successful IA MT recanalisation. They found that IA rt-PA can significantly increase the proportion of 90-day mRS 0–1 compared with placebo (59.0% vs 40.4%, p=0.047), without increasing the risk of sICH and reducing the 90-day all-cause mortality. However, the CHOICE study was terminated early due to slow enrolment and drug supply problems caused by the COVID-19 pandemic. Large RCTs are still needed to verify their findings.^87^		
Reworded	Patients with indications for IA MT should undergo treatment as soon as possible. When meeting the criteria for intravenous thrombolysis, intravenous thrombolysis should be initiated first while simultaneously considering bridging to IA MT.	I	A
Reworded	For patients with contraindications to intravenous thrombolysis, it is recommended considering direct IA MT as the treatment option for eligible patients with LVO.	IIa	A
Reworded	For patients with occlusion in the MCA M2 or M3 segments, IA MT may be considered if the onset of symptoms is within 6 hours.	IIb	B
Reworded	The benefit of MT in patients with AIS with LVO beyond 24 hours of onset is uncertain.	IIb	C

### Section 4: antiplatelet therapy for acute ischaemic cerebrovascular disease

**Figure 6:**
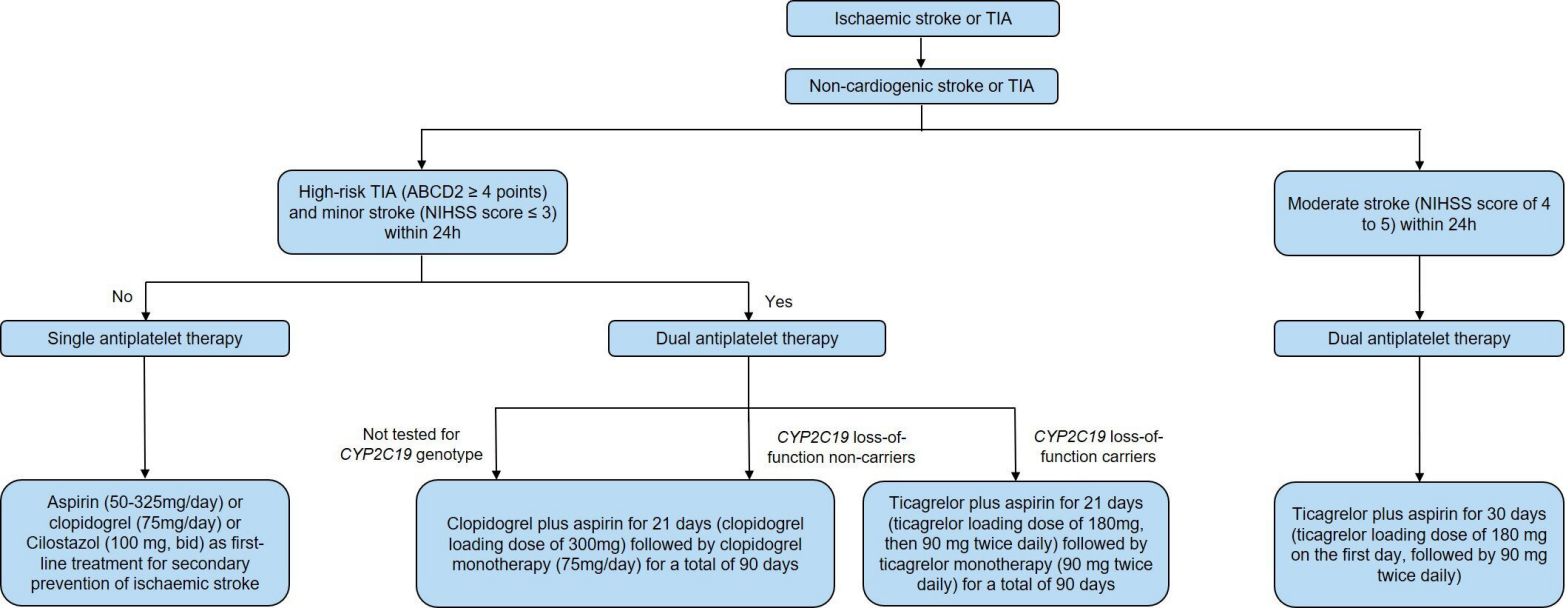
The antiplatelet treatment process for patients with acute ischaemic stroke (AIS). bid, two times per day; NIHSS, National Institutes of Health Stroke Scale; TIA, transient ischaemic attack.

The antiplatelet treatment process for patients with AIS is shown in figure 6.

#### Section 4.1 Single antiplatelet therapy

**Table:** 

Table 4.1 Single antiplatelet therapy		COR	LOE
Reworded	Patients with AIS are recommended to take aspirin within 24–48 hours after the onset of symptoms. For patients undergoing intravenous thrombolysis, the administration of aspirin is typically delayed until 24 hours after treatment.	I	A
Reworded	Monotherapy with either aspirin (50–325 mg/day) or clopidogrel (75 mg/day) can be considered the preferred antiplatelet treatment option for secondary stroke prevention.	I	A
Reworded	Ticagrelor monotherapy (as a substitute for aspirin monotherapy) is not recommended for the acute treatment of minor stroke and TIA.	III	B
New recommendation	Cilostazol is an alternative treatment to aspirin and clopidogrel for patients with AIS who are at high risk of bleeding.	IIb	B
	The CASISP study showed that both aspirin and cilostazol could reduce stroke recurrence rates, with no significant difference between the two groups (HR 0.62, 95% CI 0.30 to 1.26, p=0.185).^88^ However, cilostazol showed a lower incidence of bleeding complications than aspirin (1 vs 7, p=0.034). It makes cilostazol a potentially suitable choice for Chinese patients with IS who have a higher risk of bleeding. The CSPS II study found that in Japanese patients with non-cardioembolic IS, the cilostazol group had a significantly lower annual stroke recurrence rate compared with the aspirin group (2.76% vs 3.71%, relative risk reduction (RRR) 25.7%, p=0.0357).^89^ Furthermore, the cilostazol group demonstrated a reduced annual incidence of haemorrhagic stroke or haemorrhage necessitating hospitalisation compared with the aspirin group (0.77% vs 1.77%, RRR 54.2%, p=0.0004). However, limited research from non-Asian populations is available to confirm these findings. Therefore, the generalisability of these results to non-Asian populations may require further investigation.		
Revised	For patients with moderate-to-severe IS, it is not recommended using indobufen for secondary stroke prevention.	III	B
	The INSURE trial found that compared with aspirin, indobufen did not exhibit a significant reduction in the risk of stroke recurrence (HR 1.23, 95% CI 1.01 to 1.50, p for non-inferiority=0.44) or bleeding (HR 0.63, 95% CI 0.35 to 1.15, p=0.13) at 90 days.^90^		
Reworded	Abciximab is not recommended for AIS.	III	B
New recommendation	For patients with AIS, receiving intravenous tirofiban before IA MT is not recommended.	III	B
	The RESCUE BT trial found that compared with placebo, tirofiban did not reduce the risk of disability at 90 days (adjusted OR 1.08, 95% CI 0.86 to 1.36).^91^		
New recommendation	Intravenous tirofiban can be beneficial in those patients who meet the RESCUE BT2 trial inclusion criteria.	IIb	B
	The RESCUE BT2 trial found that administering intravenous tirofiban to a heterogeneous patient group with recent onset or progression of stroke symptoms and non-occluded large and medium-sized cerebral vessels resulted in a higher likelihood of excellent outcomes than low-dose aspirin (adjusted RR 1.26, 95% CI 1.04 to 1.53).^92^		

#### Section 4.2 Dual antiplatelet therapy

**Table:** 

Table 4.2 Dual antiplatelet therapy		COR	LOE
Reworded	For patients with minor IS and high-risk TIA who did not receive intravenous thrombolysis, dual antiplatelet therapy is initiated within 24 hours of symptom onset if their NIHSS score is <3. The recommended treatment regimen includes aspirin 100 mg/day and clopidogrel 75 mg/day (with a loading dose of 300 mg on the first day) for 21 days. Subsequently, the treatment can be switched to monotherapy with clopidogrel 75 mg/day for 90 days.	I	A
New recommendation	For patients with non-cardioembolic minor IS (NIHSS score ≤3) or high-risk TIA (ABCD2 score ≥4) who present within 24 hours of symptoms onset, it is recommended considering *CYP2C19* genetic rapid testing to determine if the patient carries *CYP2C19* LoF alleles. The results of this genetic testing will aid in selecting antiplatelet agents.	I	A
	The genetic subgroup analysis of the CHANCE study revealed that patients carrying *CYP2C19* LoF alleles did not show a significant reduction in stroke recurrence when treated with clopidogrel plus aspirin compared with aspirin alone.^93^ There was an interaction between the treatment group and genetic variation. The PRINCE study found that in patients who had a stroke or TIA carrying *CYP2C19* LoF alleles, early administration of ticagrelor with aspirin reduced platelet hyper-reactivity and lowered the risk of 90-day stroke recurrence (secondary outcome) compared with clopidogrel with aspirin.^94^		
New recommendation	For patients with non-cardioembolic minor IS (NIHSS score ≤3) or high-risk TIA (ABCD2 score ≤4) who present within 24 hours of symptom onset, if *CYP2C19* gene can be tested and the patient carries *CYP2C19* LoF alleles, ticagrelor plus aspirin for 21 days (ticagrelor loading dose of 180 mg on the first day, followed by 90 mg two times per day), and continue with ticagrelor monotherapy (90 mg two times per day) for 90 days are recommended.	I	A
	The CHANCE-2 study found that among patients with minor stroke or high-risk TIA who carry *CYP2C19* LoF (*2, *3) alleles, ticagrelor plus aspirin demonstrated superior efficacy in preventing stroke recurrence compared with clopidogrel plus aspirin (HR 0.77, 95% CI 0.64 to 0.94, p=0.008).^6^ The PRINCE study found that in patients who had a stroke or TIA carrying *CYP2C19* LoF alleles, early administration of ticagrelor with aspirin reduced platelet hyper-reactivity and lowered the risk of 90-day stroke recurrence (secondary outcome) compared with clopidogrel with aspirin.^94^		
New recommendation	For patients with moderate IS (NIHSS score of 4–5) who present within 24 hours of symptom onset, ticagrelor plus aspirin for 30 days (ticagrelor loading dose of 180 mg on the first day, followed by 90 mg two times per day) may reduce the risk of recurrent stroke and death within 30 days.	IIb	B
	The THALES study found that patients with moderate stroke (NIHSS score of 4–5) benefited from ticagrelor plus aspirin compared (NIHSS score ≤3) with aspirin alone, as opposed to patients with minor strokes, without an increased risk of intracranial bleeding or other severe bleeding events.^95^		

#### Section 4.3 Triple antiplatelet therapy

**Table:** 

Table 4.3 Triple antiplatelet therapy		COR	LOE
Reworded	Triple antiplatelet therapy (aspirin, clopidogrel and dipyridamole) is not recommended and may be harmful.	III	B

### Section 5: other treatments in the acute phase

#### Section 5.1 Brain cytoprotection

**Table:** 

Table 5.1 Brain cytoprotection		COR	LOE
New recommendation	Brain cytoprotection with edaravone dexborneol (intravenous 37.5 mg/dose, once every 12 hours, for 14 days) may improve clinical outcomes in patients with AIS.	IIa	B
	Preclinical and clinical studies suggest that edaravone can reduce the incidence of complications such as haemorrhagic transformation, progressive stroke, epilepsy and pulmonary infection in patients with IS. The RESCUE-Japan Registry study subgroup analysis found that the combination of intravenous rt-PA with edaravone was more effective in obtaining favourable outcomes in patients with AIS with LVO.^96^ Edaravone dexborneol, comprised of two active ingredients, edaravone and (+)−borneol, has been developed as a novel neuroprotective agent with synergistic antioxidant and anti-inflammatory effects in animal models. The TASTE study is a multicentre, randomised, double-blind, comparative, phase III clinical trial conducted at 48 hospitals in China to investigate the effects of edaravone dexborneol versus edaravone on 90-day functional outcomes in patients with AIS. The results indicated that when edaravone dexborneol versus edaravone was administered within 48 hours after AIS, 90-day good functional outcomes favoured the edaravone dexborneol group, especially in female patients (mRS score ≤1, 67.18% vs 58.97%, OR 1.42, 95% CI 1.12 to 1.81, p=0.004).^97^		
New recommendation	NBP, 25 mg, dissolved in 100 mL sodium chloride and given as intravenous injection two times per day for the first 14 days, followed by soft 0.2 g capsules of NBP three times a day for the next 76 days, may serve as an adjunct treatment to reperfusion therapy and have the potential to improve functional outcomes in patients with AIS.	IIb	B
	The BAST trial, a multicentre, double-blind, placebo-controlled, parallel randomised clinical trial, aimed to investigate whether treatment with NBP adjunctive to reperfusion therapy of intravenous thrombolysis and/or IA MT could improve the functional outcome in patients with AIS compared with placebo. The results showed that the proportion of patients achieving a favourable outcome based on the 90-day mRS score was significantly higher in the NBP group compared with the placebo group (56.7% vs 44.0%, OR 1.70, 95% CI 1.35 to 2.14, p<0.001). The rate of serious adverse events was similar between the two groups.^98^		

#### Section 5.2 Brain cytoprotection

**Table:** 

**Table 5.2 Hypothermia**				COR	LOE
Reworded	The efficacy and safety of induced hypothermia in patients with AIS are unclear, and further studies are needed. Most studies indicate an association between induced hypothermia and increased risk of infection, including pneumonia. Induced hypothermia should be administered only in clinical trials.	IIb	B

#### Section 5.3 Hyperbaric oxygen therapy

**Table:** 

**5.3 Hyperbaric oxygen therapy**				COR	LOE
Unchanged	Hyperbaric oxygen therapy is not recommended for patients with AIS unless caused by air embolism. Hyperbaric oxygen therapy is related to claustrophobia, middle ear barotrauma and the increased risk of seizures.	III	B
**5.4 Mechanical augmentation of blood flow**			
Unchanged	Mechanical augmentation of blood flow to treat patients with AIS has not been perfected. The curative effect is not sure and only can be used in clinical trials.	IIb	B
**5.5 Blood volume expansion and defibrinogen therapy**			
Unchanged	It is not recommended for routine use of blood volume expansion or haemodilution therapy in patients with AIS.	III	A
Unchanged	In patients with AIS with hyperfibrinogenaemia, the effectiveness of defibrinogen therapy remains to be determined.	IIb	B
**5.6 Neural regulation therapy**			
New recommendation	Remote ischaemic conditioning may be controversial for some patients with AIS.	IIb	B
	Applying an ischaemic stimulus distant from the brain (remote ischaemic conditioning, for example, transient limb ischaemia) after a stroke can induce neuroprotection. The RECAST was a pilot blinded placebo-controlled trial in 26 patients with AIS, randomised 1:1 to receive four cycles of remote ischaemic conditioning within 24 hours of ictus. The results of the study found that remote ischaemic conditioning after acute stroke was well tolerated and appeared safe and feasible. The NIHSS score was significantly reduced at 90 days in the treatment group (median NIHSS score 1 (IQR 0.5–5) vs 3 (IQR 2–9.5), p=0.04).^99^ The RICAMIS trial, including 1893 patients with moderate AIS, was conducted at 55 hospitals in China to assess the efficacy of remote ischaemic conditioning. Treatment with remote ischaemic conditioning compared with usual care significantly increased the likelihood of excellent neurological function at 90 days (67.4% vs 62%, risk difference 5.4%, 95% CI 1.0% to 9.9%, OR 1.27, 95% CI 1.05 to 1.54, p=0.02).^100^ The REPOST study was a randomised, single-blind, placebo-controlled clinical trial. Eighty-eight patients with IS in the past 24 hours were randomised 1:1 to repeated remote ischaemic postconditioning (rIPostC) or sham conditioning. The study found no significant improvement in infarct size or clinical outcome in patients with AIS treated with repeated rIPostC.^101^ However, the inclusion rate was lower than expected, and no definite conclusions about the effectiveness of rIPostC could be drawn.		
Revised	There is no evidence that transcranial near-infrared laser therapy for IS is beneficial. Therefore, using transcranial near-infrared laser treatment in IS is not recommended.	III	B
	Previous data suggested that transcranial near-infrared laser therapy for stroke held promise as a therapeutic intervention through data published in the NEST-1 and NEST-2 trials.^102–104^ The NEST-3 trial examined the application in patients with moderate stroke (NIHSS score 7–17) who did not receive intravenous rt-PA.^105^ The study was halted due to futility after analysing the first 566 patients, as no benefit of transcranial laser therapy over sham treatment was observed. Currently, there is no evidence supporting the efficacy of transcranial laser therapy in treating IS.		
New recommendation	Transcranial magnetic stimulation therapy may contribute to motor and cognitive function recovery in patients with AIS.	IIb	C
	Two RCTs have ascertained the efficacy of low-frequency transcranial magnetic stimulation (TMS) therapy in ameliorating motor function deficits and cognitive impairments. However, these trials had limited sample sizes.^106 107^ Given the variations in stimulation parameters across studies, conducting additional comprehensive clinical trials is essential to refine the optimal treatment parameters for TMS.		
**5.7 High-dose albumin therapy**			
Unchanged	The routine use of high-dose albumin therapy in patients with AIS is not recommended.	III	A

### Section 6: general supportive treatment and management of complications

**Figure 7:**
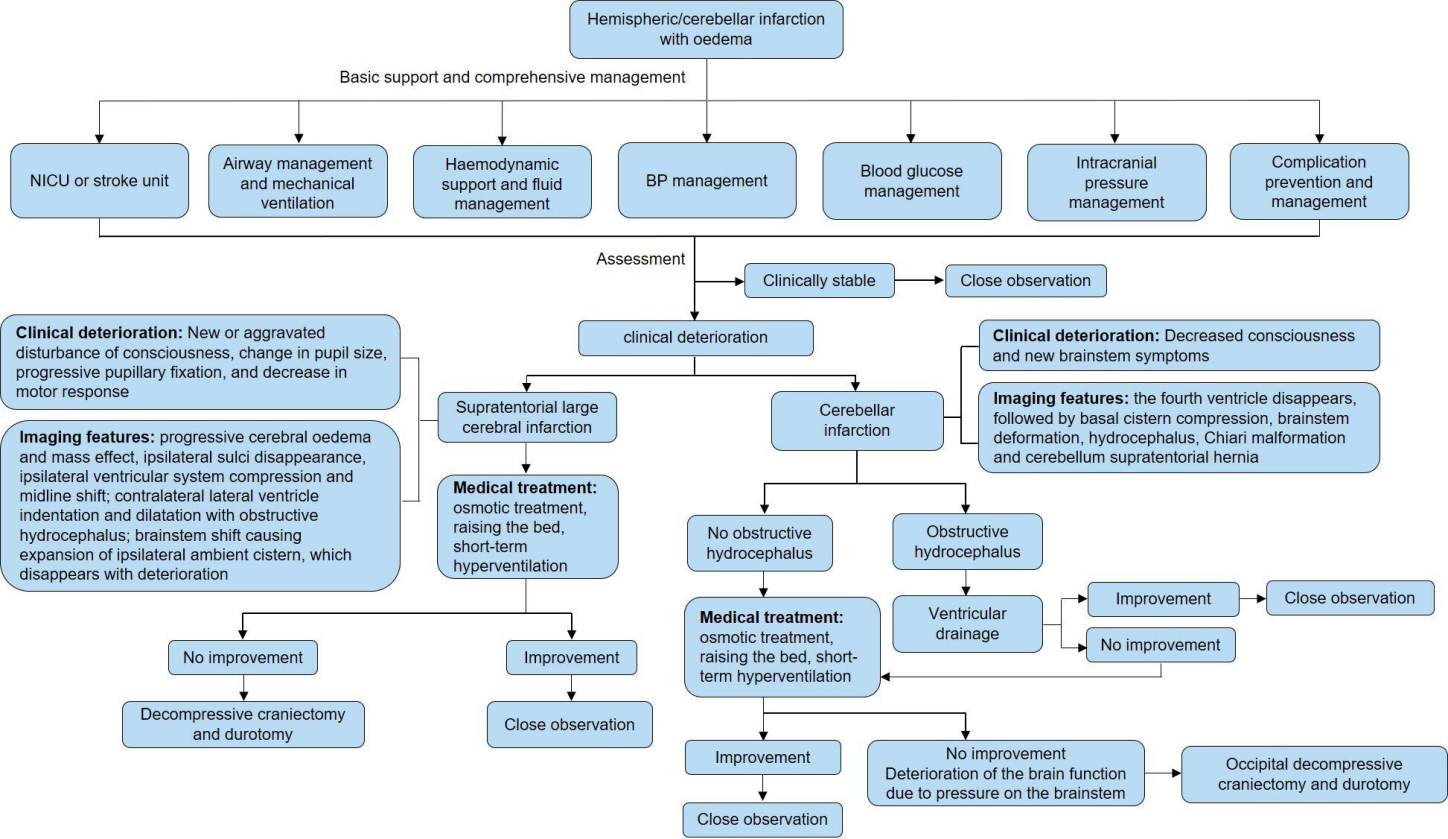
The management process for brain oedema/intracranial hypertension. BP, blood pressure; NICU, neurological intensive care unit.

**Figure 8:**
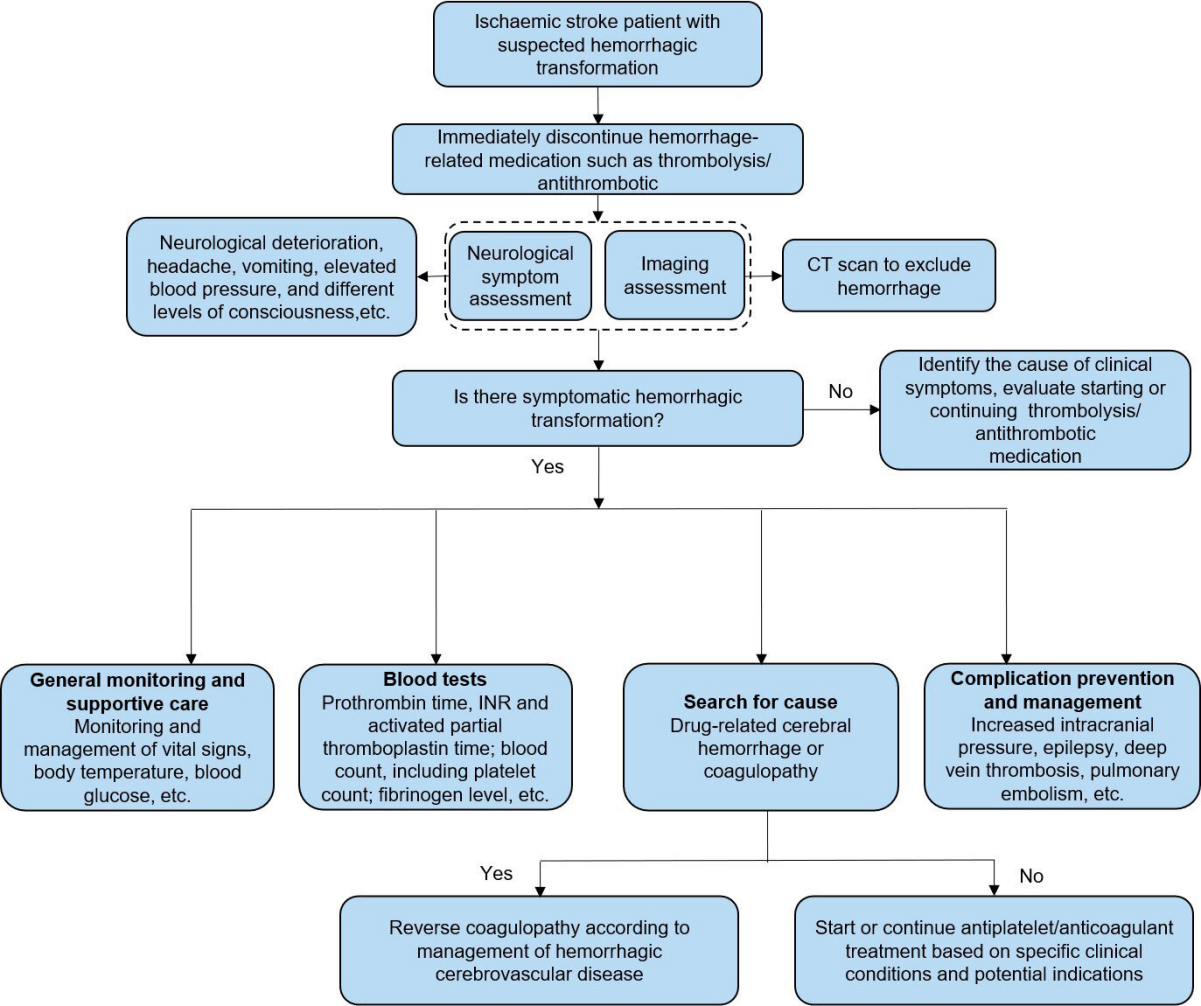
The management process for haemorrhagic transformation in patients with acute ischaemic stroke. INR, international normalised ratio.

**Figure 9:**
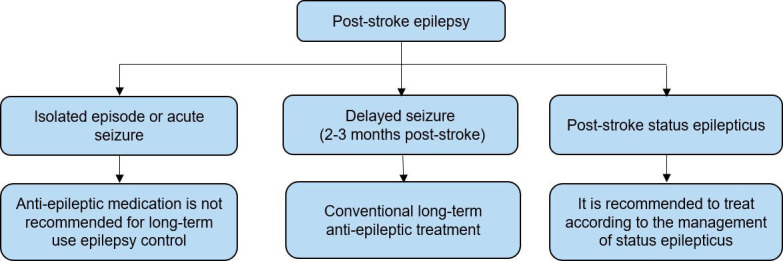
The management process for the first seizure within 24 hours of stroke onset.

The management process for brain oedema/intracranial hypertension is shown in figure 7. The management process for haemorrhagic transformation in patients with AIS is shown in figure 8. The management process for the first seizure within 24 hours of stroke onset is shown in figure 9.

**Table:** 

6.1 General supportive care		COR	LOE
6.1.1 Airway support, ventilator assistance and supplemental oxygen			
Unchanged	Airway support and ventilator assistance are recommended for the treatment of patients with AIS who have decreased consciousness or who have bulbar dysfunction that causes compromise of the airway.	I	C
Unchanged	Supplemental oxygen should be provided to maintain oxygen saturation >94%.	I	C
Unchanged	Supplemental oxygen is not recommended in non-hypoxic patients with AIS.	III	B
6.1.2 Body temperature			
Reworded	The cause of fever (body temperature >38°C) should be investigated. Pharmacological antipyretic therapy should be administered to patients who had a stroke who have a fever.	I	C
6.1.3 Nutrition			
Reworded	Enteral nutrition should be initiated within 7 days of hospitalisation for patients with AIS.	I	B
Reworded	For patients with dysphagia, nasogastric tube feeding should be provided during the early stages of stroke (within 7 days of onset). Percutaneous gastrostomy tube placement is considered appropriate when anticipated dysphagia is expected to persist for an extended period (more than 2–3 weeks).	IIa	C
Reworded	Nutritional supplements are reasonable for patients who are malnourished or at risk of malnourishment.	IIa	B
6.1.4 Dysphagia			
New recommendation	Dysphagia screening before the patient begins eating, drinking or receiving oral medications is reasonable, which may help to identify patients at high risk of aspiration.	I	C
	Dysphagia, a common (37–78%) complication of acute stroke, is a risk factor for aspiration pneumonia and is associated with higher mortality and worse patient outcomes. The Evidence Review Committee completed a systematic review to determine whether dysphagia screening, compared with no screening or usual care, decreased outcomes of pneumonia, death or dependency.^108–111^ There were insufficient data to determine whether implementing a dysphagia screening protocol reduces the risk of death or dependency. Previous studies found that patients who failed dysphagia screening were older, had a higher rate of multiple comorbidities (including prior stroke and dementia), more often came from a long-term care facility, more often presented with weakness and speech deficits, had a lower level of consciousness and had a higher stroke severity.^112^ Besides, patients who failed dysphagia screening were more likely to develop pneumonia (13.1% vs 1.9%), to have a more severe disability (52.4% vs 18.0%) and to be discharged to a long-term care institution (14.0% vs 4.3%). Early dysphagia screening can effectively identify patients at higher risk of aspiration, which is associated with a greater risk of pneumonia, even if dysphagia screening was not associated with reduced rates of pneumonia or improvements in death or disability when tested in RCTs.^109–111^		
New recommendation	An endoscopic evaluation is reasonable for those patients suspected of aspiration to verify the presence/absence of aspiration and to determine the physiological reasons for dysphagia to guide the treatment plan.	IIa	B
	Instrumental evaluation (videofluoroscopy, fibreoptic endoscopic evaluation of swallowing or fibreoptic endoscopic evaluation of swallowing with sensory testing) allows the clinician to visualise swallow physiology, thus determining the presence or absence of aspiration, the quantity of aspiration, and the physiological or structural causes for dysphagia. This information is necessary for forming an appropriate and effective treatment plan, including swallow therapy and diet recommendations.^113–115^		
New recommendation	It is reasonable for dysphagia screening to be performed by a speech/language pathology specialist or other trained healthcare providers.	IIa	C
	Three RCTs evaluated computer-based therapy: one against no treatment, another against the same treatment provided by a speech and language therapist, and a third against non-linguistic computer training.^116–118^ These three trials concluded that computer-based therapy is feasible and efficacious. Therefore, computerised treatment is beneficial and can be used to supplement treatment provided by a speech/language pathologist.		
New recommendation	It is not well established which instrument to choose for evaluation of swallowing with sensory testing. The choice may be based on instrument availability or other considerations (ie, fibreoptic endoscopic evaluation of swallowing, videofluoroscopy, fibreoptic endoscopic evaluation with sensory testing).	IIb	C
	There is no consensus in the literature on a preferred instrumental study. Both videofluoroscopy and fibreoptic endoscopic evaluation of swallowing can be used to evaluate the swallow mechanism. Additionally, a large cohort study showed that fibreoptic endoscopic evaluation of swallowing with sensory testing was a relatively safe procedure for evaluating the sensory and motor aspects of dysphagia. Clinical judgement should be used to weigh the advantages and disadvantages of each study for each patient.^119^		
Reworded	Maintaining oral hygiene may be a reasonable intervention to reduce the risk of post-stroke pneumonia.	IIb	B
6.1.5 Prediction of infections			
Reworded	Routine use of prophylactic antibiotics has no benefits.	III	B
Reworded	Routine placement of indwelling catheters is not recommended due to the potential increased risk of catheter-associated urinary tract infections.	III	C
New recommendation	Performing early swallowing function assessment and training for all patients with stroke can reduce the incidence of stroke-associated pneumonia.	I	B
	A formal dysphagia screen is associated with a higher adherence rate to dysphagia screens and a significantly decreased risk of pneumonia.^120^		
6.1.6 Deep vein thrombosis (DVT) prophylaxis			
Reworded	For patients who had a stroke with limited mobility and no contraindications, in addition to conventional treatment (aspirin and fluid therapy), intermittent pneumatic compression is recommended to reduce the risk of DVT.	I	B
Reworded	For patients with AIS with limited mobility, the benefit of prophylactic dose of subcutaneous heparin (unfractionated heparin (UFH) or LMWH) for DVT prophylaxis is not well established.	IIb	A
Reworded	The benefit-to-risk comparison between prophylactic doses of UFH and LMWH remains unclear.	IIb	B
Reworded	For patients with IS, elastic compression stockings are not routinely recommended.	III	B
New recommendation	The benefit of using rivaroxaban up to 45 days after discharge to prevent venous thromboembolism (VTE) for patients with AIS is unclear.	III	B
	In the randomised, double-blind MARINER trial, medically ill patients who were at an increased risk of VTE based on a modified IMPROVE score of 4 or higher or a score of 2 or 3 plus a plasma D-dimer level of more than twice the upper limit of the normal range were assigned at hospital discharge to either once-daily rivaroxaban at a dose of 10 mg (with the dose adjusted for renal insufficiency) or placebo for 45 days.^121^ The results showed that rivaroxaban, given to medical patients for 45 days after hospital discharge, was not associated with a significantly lower risk of symptomatic VTE and death due to VTE than placebo (0.83% vs 1.10%, HR 0.76, 95% CI 0.52 to 1.09, p=0.14). The incidence of major bleeding was low.		
New recommendation	Prolonging venous thromboprophylaxis for 4–5 weeks for patients with AIS can reduce the risk of VTE.	IIa	A
	A 2020 meta-analysis for RCTs comparing extended versus standard venous thromboprophylaxis in patients hospitalised for AIS indicates that VTE risk was lower in patients with AIS receiving extended thromboprophylaxis (RR 0.67, 95% CI 0.43 to 1.04, 13 fewer per 1000), whereas the increase in major bleeding seemed trivial when compared with standard prophylaxis (RR 1.10, 95% CI 0.31 to 3.95, 1 more per 1000).^122^ The net clinical benefit may favour extended venous thromboprophylaxis for 4–5 weeks over standard thromboprophylaxis.		
6.1.7 Rehabilitation			
Reworded	It is recommended providing early rehabilitative therapy within an organised interdisciplinary stroke care environment for patients who had a stroke.	I	A
Unchanged	It is recommended that stroke survivors receive rehabilitation at an intensity commensurate with anticipated benefit and tolerance.	I	B
Unchanged	High-dose, very early mobilisation within 24 hours of stroke onset should not be performed.	III	B
Reworded	It is recommended that all individuals with stroke be provided with a formal assessment of their activities of daily living and instrumental activities of daily living, communication abilities and functional mobility before discharge, and the findings be incorporated into the care transition and the discharge planning process.	I	B
Unchanged	A functional assessment by a clinician with expertise in rehabilitation is recommended for patients who had a stroke with residual functional deficits.	I	C
**6.2 Management of neurological complications**			
6.2.1 Brain oedema and occupation signs			
Reworded	Patients with a large cerebral infarction are at high risk of developing brain oedema and increased intracranial pressure. Transferring these patients to the intensive care unit and implementing measures to mitigate the risk of oedema in the early post-stroke period are recommended. Close monitoring for signs of neurological deterioration in patients is crucial.	I	C
Reworded	In patients with unilateral MCA infarction, aged ≤60 years, who experience neurological deterioration within 48 hours despite receiving medical treatment, decompressive craniectomy with durotomy is reasonable.	IIa	A
Reworded	In patients with unilateral MCA infarction, aged >60 years, who experience neurological deterioration within 48 hours despite receiving medical treatment, decompressive craniectomy with durotomy may be considered.	IIb	B
Reworded	Although the optimal trigger for decompressive craniectomy is unknown, it is reasonable to consider the decline in consciousness caused by brain oedema as a criterion for surgical intervention.	IIa	A
Reworded	Ventriculostomy is recommended in the treatment of obstructive hydrocephalus after a cerebellar infarct. The decision to perform concurrent or subsequent decompressive craniectomy should be based on factors such as infarct volume, neurological condition, degree of brainstem compression and effectiveness of medical treatment.	I	C
Unchanged	Decompressive suboccipital craniectomy with durotomy should be performed in patients with cerebellar infarction causing neurological deterioration from brainstem compression despite maximal medical therapy. When deemed safe and indicated, obstructive hydrocephalus should be treated concurrently with ventriculostomy.	I	B
Unchanged	The use of salvage osmotic therapy for patients with clinical deterioration from occupying signs associated with large supratentorial infarction or cerebellar infarction is reasonable.	IIa	C
Reworded	Transient moderate hyperventilation (partial pressure of carbon dioxide target 30–34 mm Hg) as a bridging therapy is an appropriate treatment for patients with the acute severe neurological decline due to brain oedema.	IIa	C
Reworded	For patients with cerebral hemisphere or cerebellar infarction accompanied by brain oedema, using hypothermia or barbiturate drugs is not recommended.	III	B
Reworded	Because of a lack of evidence of efficacy and the potential to increase the risk of infectious complications, corticosteroids (in conventional or large doses) should not be administered to treat brain oedema and increased intracranial pressure.	III	A
6.2.2 Haemorrhagic transformation			
Unchanged	Symptomatic haemorrhagic transformation: stop using antithrombotic (antiplatelet, anticoagulation); for haemorrhage management associated with anticoagulation and thrombolysis, refer to cerebral haemorrhage treatment guidelines.	I	C
Unchanged	For patients with AIS with haemorrhagic transformation, starting or continuing antiplatelet or anticoagulant therapy should only be decided according to the specific clinical conditions and potential indications.	IIb	B
6.2.3 Seizures after AIS			
Reworded	The treatment of recurrent seizures following stroke should be similar to the management of seizures following other acute neurological conditions, with the selection of antiepileptic drugs based on individual patient characteristics.	I	C
New recommendation	In patients with epilepsy after IS, lamotrigine or levetiracetam monotherapy may be preferable to carbamazepine or sodium valproate monotherapy.	IIb	B
	Recently, a cohort study investigated whether mortality varies with specific anti-seizure medication among patients with post-stroke epilepsy.^123^ Based on individual-level data from linked registers on all adults in Sweden, the study included 2577 patients receiving continuous anti-seizure medication monotherapy. The dispensed anti-seizure medication determined exposure status, and the first dispensation date marked the start of treatment. The primary outcome was all-cause death analysed using Cox proportional hazards regression with carbamazepine as the reference. This cohort study’s findings suggest differences in survival between patients treated with different anti-seizure medications for post-stroke epilepsy. Patients receiving lamotrigine monotherapy had significantly lower mortality (adjusted HR 0.76, 95% CI 0.61 to 0.95) than those receiving carbamazepine. The opposite applied to patients prescribed valproic acid, with a higher risk of cardiovascular and all-cause death (adjusted HR 1.40, 95% CI 1.19 to 1.64). Levetiracetam was associated with a reduced risk of cardiovascular death compared with carbamazepine (adjusted HR 0.77, 95% CI 0.60 to 0.99). However, there was no significant difference in overall mortality.		
New recommendation	The SeLECT score is recommended to predict the risk of late seizures after IS.	I	B
	The SeLECT is an easily applied instrument to predict late (>7 days) seizures after IS.^124^ The score consists of five variables: severity of stroke, large-artery atherosclerotic aetiology, early seizures, cortical involvement and territory of MCA involvement. The SeLECT score was developed based on five clinical predictors in 1200 participants who had an IS in Switzerland using backward elimination of a multivariable Cox proportional hazards model and externally validated in 1169 participants from three independent international cohorts in Austria, Germany and Italy, and assessed its performance with the concordance statistic and calibration plots. The lowest SeLECT value (0 points) was associated with a 0.7% (95% CI 0.4% to 1.0%) risk of late seizures within 1 year after stroke (1.3% (95% CI 0.7% to 1.8%) within 5 years), whereas the highest value (9 points) predicted a 63% (42% to 77%) risk of late seizures within 1 year (83% (62% to 93%) within 5 years). The model had an overall concordance statistic of 0.77 (95% CI 0.71 to 0.82) in the validation cohorts. Calibration plots indicated high agreement between predicted and observed outcomes.		
New recommendation	Prophylactic use of anti-seizure drugs is not recommended.	III	C
	No studies to date have demonstrated the benefit of prophylactic anticonvulsant use after IS.		

### Section 7: early evaluation of aetiology and pathogenesis of ischaemic cerebrovascular disease

**Figure 10:**
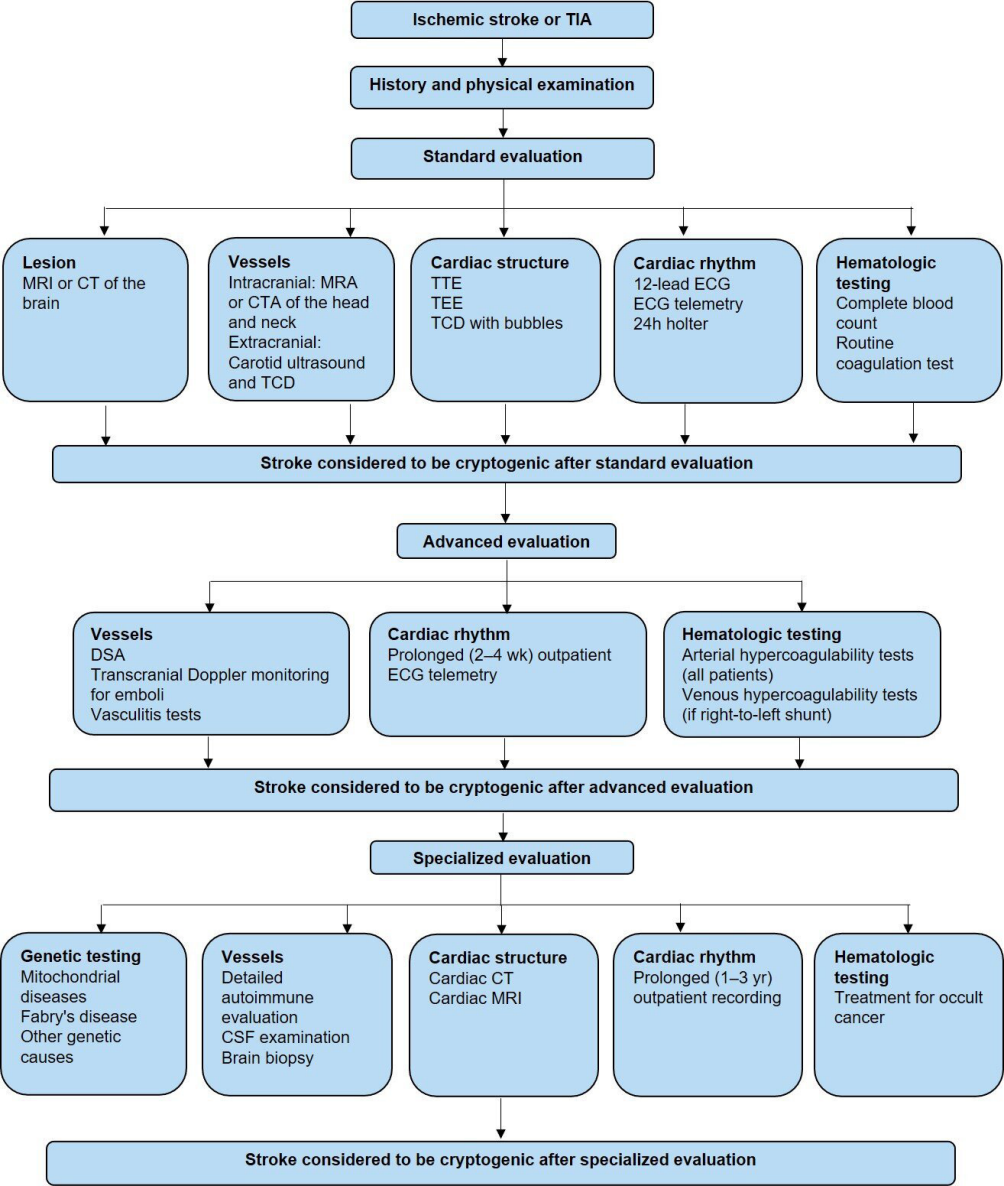
The diagnostic process for cryptogenic stroke. CSF, cerebrospinal fluid; CTA, CT angiography; DSA, digital subtraction angiography; MRA, magnetic resonance angiography; TCD, transcranial Doppler; TEE, transoesophageal echocardiography; TIA, transient ischaemic attack; TTE, transthoracic echocardiography.

The diagnostic process for cryptogenic stroke is shown in figure 10.

**Table:** 

7.1 Recommendations for completing examinations and assessments		COR	LOE
Reworded	All patients with stroke are recommended undergoing routine chest X-rays and transthoracic echocardiography to search for possible cardiac structural diseases.	I	C
Reworded	For patients who had a stroke with suspected embolic aetiology, performing transoesophageal echocardiography to look for left atrial appendage thrombus, patent foramen ovale (PFO) or atrial septal aneurysm is reasonable.	IIa	B
Unchanged	Transthoracic echocardiography cannot be replaced by transoesophageal echocardiography.	III	C
Reworded	Cardiac MRI is effective in identifying the aetiology of cryptogenic stroke. It is recommended performing when available.	I	A
Reworded	Cardiac abnormalities detected during cardiovascular screening in patients who had a stroke should be actively managed under the guidance of a specialist physician.	I	B
New recommendation	In patients suspected of having a PFO-related stroke, transcranial Doppler (TCD) with bubbles might be reasonable to screen for the presence of a right-to-left shunt.	I	B
	TCD compares favourably with transthoracic echocardiography for detecting right-to-left shunting, which is usually the result of PFO, now a potential target for device closure.^125^ A pooled analysis of the Oxford Vascular Study data with data from two previous smaller studies of bubble TCD in patients ≥50 years of age found an association between right-to-left shunting and cryptogenic TIA or non-disabling stroke (OR 2.35, 95% CI 1.42 to 3.90). A pooled analysis of a systematic literature review found that TCD had a sensitivity of 96.1% (95% CI 93.0% to 97.8%) and specificity of 92.4% (95% CI 85.5% to 96.1%) compared with transthoracic echocardiography (gold standard) for detection of right-to-left shunting.^126^		
New recommendation	For patients with IS, it is recommended performing a 12-lead ECG to screen for atrial fibrillation and atrial flutter, and assess for other concurrent cardiac conditions.	I	B
	The 12-lead ECG is a simple, non-invasive means of diagnosing atrial fibrillation in patients with acute stroke. A meta-analysis found that the proportion of patients diagnosed with post-stroke atrial fibrillation in the emergency department by ECG was 7.7% (95% CI 5.0% to 10.8%). ECG can also detect pertinent comorbidities that may have therapeutic implications. About 3% of patients presenting with acute stroke also have an acute MI.		
Reworded	It is advisable to conduct routine pulse examinations for patients aged >65 years and perform a 12-lead ECG for those with abnormal findings.	I	A
Reworded	For patients with persistent atrial fibrillation, Congestive heart failure, Hypertension, Age >75, Diabetes mellitus, and prior Stroke or TIA (CHADS2) or Congestive heart failure, Hypertension, Age ≥75, Diabetes mellitus, prior Stroke or TIA, Vascular disease, Age 65–74, sex category (CHA2DS2-VASc) score is recommended to assess for their stroke risk and guide the management.	I	A
Reworded	It is reasonable to use outpatient mobile long-term telemetry, implantable loop recorders or other methods for ≥24 hours of long-term cardiac monitoring in patients with potential cryptogenic stroke, for the purpose of detecting any paroxysmal atrial fibrillation or atrial tachycardia.	IIa	B
Unchanged	For patients with non-persistent atrial fibrillation or paroxysmal atrial fibrillation/atrial tachycardia (>5.5 hours) within 30 days or paroxysmal atrial fibrillation for >30 s, the stroke prevention therapy is the same as those with chronic or persistent atrial fibrillation.	IIb	B
Reworded	Research suggests an association with thromboembolic events for arrhythmias other than atrial fibrillation, atrial flutter and paroxysmal supraventricular tachycardia. However, there is a lack of evidence demonstrating that interventions on these arrhythmias can reduce the occurrence of thromboembolic events. Therefore, it is recommended approaching the treatment based on the individual clinical condition.	III	C
Reworded	Reduced blood flow velocity in the left atrium, left atrial appendage and left ventricle, as well as spontaneous echocardiographic contrast in the left atrium, are independent risk factors for thrombus formation and subsequent thromboembolic events. It is necessary to investigate the underlying causes and intervene accordingly.	IIa	B

**Table:** 

7.2 Risk factor assessment and risk stratification		COR	LOE
7.2.1 Assessment of blood pressure			
Reworded	Blood pressure assessment should be conducted and strictly monitored after AIS.	I	A
Reworded	Blood pressure variability and pulse pressure have been suggested as potential factors associated with the prognosis of AIS. When monitoring blood pressure, attention should be paid to changes in these two indicators.	IIa	A
7.2.2 Evaluation of blood lipid			
Unchanged	Dyslipidaemia (too high or too low) is closely related to poor prognosis. Serum lipid levels should be actively assessed after AIS to guide lipid-lowering treatment and secondary prevention.	IIa	B
Unchanged	Relatively low blood lipids may indicate a more severe condition of cerebral infarction, and attention should be paid to the changes in the patient’s condition.	IIb	C
7.2.3 Assessment of blood glucose			
Reworded	Hyperglycaemia and blood glucose fluctuations after AIS are closely associated with stroke recurrence and poor prognosis. Strict monitoring and control of blood glucose levels in clinical practice are recommended.	IIa	B
New recommendation	It is reasonable for patients with IS or TIA to receive fasting glucose, HbA1c or oral glucose tolerance test (OGTT) screening to check for abnormal glucose metabolism after stroke. HbA1c should be used in the acute phase to screen for diabetes and pre-diabetes. Patients with no apparent history of diabetes or no precise diagnosis of diabetes should routinely receive OGTT screening for pre-diabetes and diabetes after the acute phase.	IIa	B
	Patients with abnormal glucose metabolism are at risk of diabetes and major adverse cardiovascular events. HbA1c, fasting plasma glucose and OGTT are available methods for glucose metabolism screening. HbA1c is probably the preferred diagnostic method, which is more convenient (ie, it does not require fasting) and has less variability over a short period, making it suitable for acute phase screening. OGTT can comprehensively evaluate fasting plasma glucose and 2-hour postprandial glucose, early detection of abnormal glucose metabolism and reduce the rate of missed diagnosis of diabetes.^127^		

### Section 8: interventions targeting aetiology and pathogenesis

**Figure 11:**
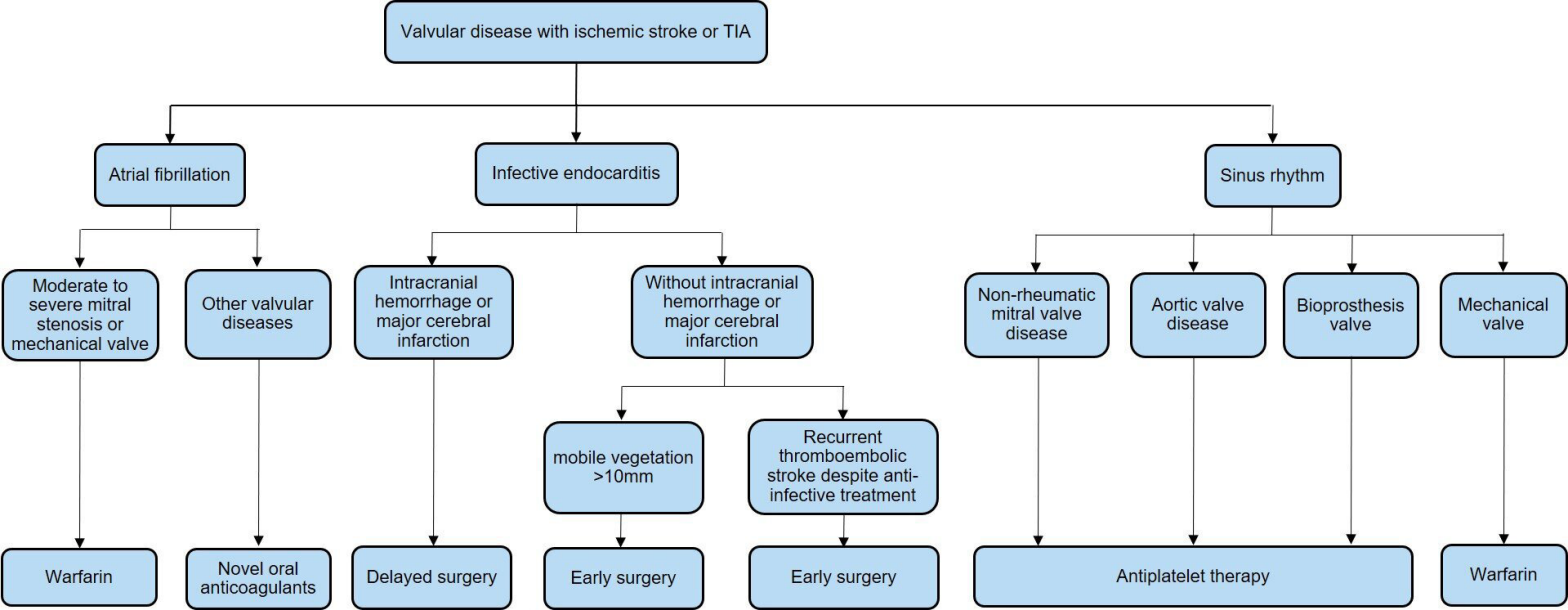
The treatment strategy of valvular heart disease. TIA, transient ischaemic attack.

The treatment strategy for valvular heart disease is shown in figure 11.

**Table:** 

8.1 Large artery atherosclerosis		COR	LOE
Unchanged	For patients with symptomatic intracranial arterial stenosis, antiplatelet is recommended over warfarin to prevent stroke and other cardiovascular events.	I	A
Unchanged	For patients with IS or TIA attributable to severe symptomatic intracranial artery stenosis (70–99%) within 30 days of onset, aspirin combined with clopidogrel is recommended for 90 days, after which aspirin or clopidogrel alone can be used as a long-term secondary prevention drug.	IIa	B
New recommendation	For patients with TIA or non-acute IS with symptomatic intracranial or extracranial arterial stenosis (50–99%) or combined with more than two risk factors, cilostazol combined with aspirin or clopidogrel may be considered.	IIb	B
	The results from CSPS.com indicated that for patients with non-acute stroke with moderate-to-severe intracranial or extracranial stenosis and two or more vascular risk factors (age ≥65 years, hypertension, diabetes, CKD, peripheral artery disease, history of IS, history of ischaemic heart disease, smoking), the combination of cilostazol with aspirin or clopidogrel reduced the risk of stroke recurrence compared with using aspirin or clopidogrel alone, without increasing the risk of any bleeding. However, in subgroup analysis, the combination of cilostazol and aspirin showed no significant difference in effectiveness and safety compared with aspirin monotherapy or dual antiplatelet therapy.^128^		
New recommendation	For patients with non-cardioembolic minor stroke (NIHSS score ≤5) or high-risk TIA (ABCD2 score ≥4) occurring within 24 hours of onset, and with mild or greater ipsilateral intracranial arterial stenosis (stenosis rate >30%), dual antiplatelet therapy with aspirin and ticagrelor (initial dose of 180 mg, followed by 90 mg two times per day) may be an option. Switching to single antiplatelet therapy is recommended after 30 days of dual antiplatelet therapy. However, clinicians should carefully balance this treatment selection’s potential benefits and bleeding risks.	IIb	B
	The results of the CHANCE-2 trial showed that in patients with minor stroke or high-risk TIA who carry the *CYP2C19* LoF, using ticagrelor instead of clopidogrel in dual antiplatelet therapy can reduce the risk of stroke recurrence at 90 days. Subgroup analysis showed that patients with symptomatic intracranial arterial stenosis had a reduction in stroke recurrence. However, the result was not significant (HR 0.76, 95% CI 0.55 to 1.04).^6 94^		
New recommendation	For symptomatic severe intracranial atherosclerotic stenosis (70–99%), percutaneous transluminal angioplasty and stenting (PTAS) should not be used as the initial treatment for such patients, even if patients are taking an antithrombotic agent at the time of stroke or TIA onset.	III	A
	Four RCTs have compared PTAS with medical treatment to prevent stroke or TIA recurrence in patients with stroke attributable to 70–99% stenosis.^129–132^ However, none of these trials have found any additional benefit of stenting over medical treatment alone.		
New recommendation	For patients with symptomatic intracranial atherosclerotic moderate stenosis (50–69%), PTAS has a higher risk of disability and death than medical treatment. Therefore, PTAS is not recommended.	III	B
	Currently, RCTs comparing the clinical outcomes of PTAS and medical therapy in patients with asymptomatic moderate intracranial arterial stenosis (50–69%) are lacking. The risk of stroke following standard medical therapy is relatively low in patients with moderate intracranial arterial stenosis, and the perioperative risk does not vary with the degree of stenosis.^133^ As RCTs have not yet demonstrated significant benefits of PTAS for patients with severe stenosis, the support for PTAS in moderate stenosis is also not endorsed.		
New recommendation	Extracranial–intracranial bypass is not recommended for patients with intracranial atherosclerotic stenosis (50–99%) or occlusion that caused stroke or TIA.	III	B
	A multicentre RCT involved 1377 patients with recent minor stroke or TIA and compared extracranial–intracranial bypass surgery with medical treatment for severe stenosis (≥70%) of the ICA or MCA.^134^ The results showed that compared with the medical treatment group, patients in the bypass surgery group had a higher proportion and earlier occurrence of fatal and non-fatal strokes.		
New recommendation	For patients with recent IS or TIA within 6 months combined with severe stenosis (70–99%) in the extracranial segment of the ipsilateral carotid artery, if the expected risk of perioperative mortality or stroke recurrence is <6%, it is recommended undergoing carotid endarterectomy (CEA) or carotid artery stenting (CAS) treatment.	I	A
	The combined analysis of the ECST, CSP and NASCET trials found that in patients with severe stenosis (70–99%) of the carotid artery, CEA had an absolute benefit of 16.0% within 5 years.^135^ Several large studies, such as the CREST trial, have confirmed no significant difference in the incidence of stroke between the CEA and CAS groups.^136–140^		
New recommendation	For patients with recent IS or TIA within 6 months combined with moderate stenosis (50–69%) in the extracranial segment of the ipsilateral carotid artery, if the expected risk of perioperative mortality or stroke recurrence is <6%, CEA or CAS is recommended. CEA or CAS should be selected according to the patient’s condition.	I	B
	The ECST trial did not observe substantial advantages in individuals with carotid artery stenosis ranging from 50% to 69%.^135 141^ Conversely, both NASCET and VA309 trials demonstrated notable and statistically significant benefits.^135 142 143^		
New recommendation	CEA or CAS is not recommended for patients with <50% stenosis in the extracranial segment of the carotid artery.	III	A
	The combined analysis of the ECST, VA309 and NASCET trials revealed that CEA showed no benefit for patients with ICA stenosis of less than 50%.^141–143^		
New recommendation	For patients who meet the indications for CEA or CAS treatment, for those aged ≥70 years, CEA is recommended over CAS. If surgery is planned within 1 week after stroke onset, CEA is also recommended over CAS.	IIa	B
	The analysis conducted by the Carotid Stenting Triallists Collaboration, which included four RCTs, found that in the age group of 65–69 years, the HR was 1.61 (95% CI 0.90 to 2.88) when comparing CAS with CEA. In the age group of 70–74 years, the HR for CAS compared with CEA was 2.09 (95% CI 1.32 to 3.32). Therefore, in patients aged 70 years and above, CEA was significantly superior to CAS in reducing the perioperative risk of stroke.^144^		
New recommendation	For patients with severe stenosis (≥70%) who meet the indications for CEA or CAS treatment, if the risk of CEA is high (such as radiation-induced stenosis or restenosis after CEA), CAS is the choice of treatment.	IIa	C
	When non-invasive imaging shows carotid artery stenosis ≥70% or DSA shows stenosis >50%, and the risk of complications from the intervention is <2%, particularly in patients with significant cardiovascular disease, CAS may be considered an alternative treatment option to CEA.	IIb	B
	In the SAPPHIRE trial, patients with higher anatomical or physiological risk for carotid revascularisation were assigned to CEA or CAS.^136^ The results showed that among symptomatic patients, 16.8% of CAS patients and 16.5% of CEA patients experienced the primary endpoint events, including stroke, MI or death (p=0.95). It confirmed that CAS could reduce stroke rates and perioperative complications within 30 days after the surgery.		
New recommendation	For patients who plan to undergo CEA or CAS, if there are no contraindications for early recanalisation, it is reasonable to proceed within 2 weeks of stroke onset.	IIa	B
	Post hoc analysis of multiple trials has found greater benefit with CEA when surgery is performed within 2 weeks after the last non-disabling ischaemic event. Therefore, if a patient is suitable for surgery, early CEA is preferred.^145^		
New recommendation	Antiplatelet, lipid-lowering and antihypertensive therapies are recommended for patients with symptomatic ICA stenosis.	I	A
	In clinical practice, it is recommended providing antiplatelet therapy, antihypertensive treatment and statin medication for patients with symptomatic carotid artery stenosis.^135^ Two different lipid profile targets were compared in a recent trial focusing on patients with a recent stroke or TIA. The study found that achieving a low-density lipoprotein level below 1.8 mmol/L was associated with a reduced incidence of vascular events.^146^		
New recommendation	The usefulness of extracranial–intracranial bypass for patients with carotid occlusion leading to TIA or ipsilateral IS is not well established.	IIb	B
	In the COSS trial, the combined endpoint events of stroke and death within 30 days and ipsilateral stroke within 2 years were 21.0% in the surgical group and 22.7% in the medical treatment group.^147^ There was no statistically significant difference between the two groups. In the recent CMOSS trial, there was no statistically significant difference observed in the composite primary outcome between the surgical group and the medical group (8.6% vs 12.3%).^148^		
New recommendation	Antiplatelet, lipid-lowering and antihypertensive therapies are recommended for patients with symptomatic vertebral artery stenosis.	I	A
	In clinical practice, it is recommended providing antiplatelet therapy, antihypertensive treatment and statin medication for patients with symptomatic vertebral artery stenosis.^145^		
New recommendation	With symptomatic extracranial vertebral atherosclerotic stenosis (50–99%), when medical treatment is ineffective, stenting may be selected in addition to the best medical management, but the effectiveness of stenting has not yet been fully confirmed.	IIb	C
	In a combined analysis of the VAST, VIST and SAMMPRIS trials, the HR for stenting compared with medical treatment was 0.63 (95% CI 0.27 to 1.46). Therefore, no significant benefit is observed for extracranial vertebral artery stenting compared with medical treatment.^145^		
New recommendation	For patients with IS or TIA caused by aortic arch atheroma, antiplatelet therapy is recommended to prevent stroke recurrence.	I	B
	The ARCH trial compared the efficacy differences between aspirin and clopidogrel versus warfarin, but the study lacked sufficient power for the primary endpoint.^149^ Therefore, the comparative benefits of these two treatments remain unknown. However, in the warfarin group, there were six cases of vascular death (3.4%), while no deaths were reported in the dual antiplatelet therapy group (p=0.013). The available evidence suggests that warfarin may not provide a clear advantage over dual antiplatelet therapy. However, it remains uncertain whether dual antiplatelet therapy surpasses single antiplatelet therapy.		
New recommendation	For patients with IS or TIA caused by aortic arch atheroma, intensive statin therapy is recommended.	I	B
	In the ARCH study, the event rate was only 20–30% of the expected rate based on observational studies with an expected rate of >12%.^149^ This could be attributed to better management of risk factors in the trial compared with historical studies. During the trial, there was an average reduction of low-density lipoprotein cholesterol (LDL-C) by approximately 40 mg/dL to 83–84 mg/dL.		
New recommendation	Stenting or surgical treatment may be considered in patients with IS or TIA with symptomatic subclavian artery stenosis (50–99%) or occlusion causing symptoms of posterior circulation ischaemia when standard medical treatment is ineffective, and there are no surgical contraindications.	IIb	C
	Currently, there is a lack of RCTs comparing endovascular treatments and surgical revascularisation methods for subclavian atherosclerotic stenosis. Previous studies indicated that the long-term patency rate after surgical revascularisation reaches 88–95%, higher than endovascular treatments (78.1–84.5%). However, it should be noted that surgical treatment carries a higher risk of trauma and invasiveness.^150–153^		
New recommendation	For patients with IS or TIA caused by stenosis of the common carotid artery or brachiocephalic trunk (50–99%), stenting or surgical treatment may be considered when medical treatment is ineffective, and there are no surgical contraindications.	IIb	C
	Currently, there is a lack of RCTs comparing endovascular treatments and surgical revascularisation methods for atherosclerotic stenosis of the common carotid artery or brachiocephalic artery. Previous studies have shown that surgical revascularisation has a higher long-term patency rate than endovascular treatments, but it is also associated with a higher risk of trauma and invasiveness.^151^		

**Table:** 

8.2 cardiogenic stroke		COR	LOE
Unchanged	For IS or TIA patients with nonvalvular atrial fibrillation, whether paroxysmal, persistent, or permanent atrial fibrillation, oral anticoagulation is recommended to reduce stroke recurrence.	I	B
New Recommendation	With IS or TIA combined with non-valvular atrial fibrillation, it is recommended to use warfarin or novel oral anticoagulants (NOACs) to prevent recurrent thromboembolic events. The target INR is between 2.0~3.0, if warfarin is prescribed.	I	A
	A meta-analysis combining the data from four trials (apixaban, dabigatran, edoxaban, and rivaroxaban) found that novel oral anticoagulation therapy had a 51% reduction in haemorrhagic stroke and a 10% reduction in mortality. The incidence of IS or systemic embolism was reduced by 19%.		
Unchanged	If anticoagulant therapy cannot be accepted for IS or TIA prevention by patients with non-valvular atrial fibrillation, aspirin alone is recommended.	I	B
Unchanged	For IS or TIA patients with non-valvular atrial fibrillation, if anticoagulant therapy cannot be accepted, aspirin combined with clopidogrel can also be selected, and the risk of bleeding should be assessed.	IIa	B
Reworded	For patients with IS or TIA combined with non-valvular atrial fibrillation, the timing of initiating anticoagulant therapy should be selected according to the severity of ischemia and the risk of haemorrhagic transformation. For patients with a high risk of ICH, anticoagulant therapy can be delayed until 14 days after onset. For patients with a low risk of ICH, anticoagulant therapy can be started within 2 to 14 days after onset to reduce the risk of stroke recurrence. Patients with TIA can initiate anticoagulant therapy after onset to reduce the risk of stroke.	IIa	C
New Recommendation	In patients with IS or TIA associated with non-valvular atrial fibrillation, who have contraindications to lifelong anticoagulation therapy but can tolerate anticoagulation for 45 days, the option of left atrial appendage closure may be considered to prevent stroke.	IIb	B
	In two RCTs (PROTECT AF and PREVAIL) and a non-randomised trial (CAP), the results showed that the Watchman device did not significantly increase the risk of thrombus formation but reduced the risk of bleeding.^154–157^		
New Recommendation	If patients with atrial fibrillation complicated with stroke or TIA are undergoing dialysis or have renal failure, either apixaban or warfarin can be used for stroke prevention.	IIb	B
	A large retrospective study matched 2351 atrial fibrillation patients on dialysis taking apixaban, with 23 172 patients taking warfarin. The study found that patients taking apixaban had a 28% lower rate of bleeding events.^158^		
New Recommendation	Patients with IS or TIA complicated with atrial flutter can follow the anticoagulant regimen for atrial fibrillation.	I	C
	Previous observational studies indicated that atrial flutter incidence is lower than atrial fibrillation. However, patients with atrial flutter have an increased risk of developing atrial fibrillation, and the probability of stroke occurrence is similar to that of atrial fibrillation.^159 160^		
Reworded	For IS or TIA patients with valvular atrial fibrillation (ie, moderate to severe mitral stenosis or mechanical heart valve disease with atrial fibrillation), it is rcommended to use warfarin to reduce the risk of stroke recurrence.	I	B
Reworded	For patients with stroke or TIA and aortic valve or nonrheumatic mitral valve disease (eg, mitral annular calcification or mitral valve prolapse) without atrial fibrillation or other indications for anticoagulation, antiplatelet therapy is recommended to reduce the risk of stroke recurrence.	I	C
New Recommendation	For patients with IS or TIA who have undergone bioprosthetic valve replacement and do not have atrial fibrillation or other indications for anticoagulation, it is recommended to use warfarin for 3–6 months after valve replacement, followed by long-term use of aspirin.	I	C
	This recommendation is based on expert opinions and clinical practice. Some studies have reported an increased risk of IS in patients undergoing bioprosthetic valve replacement.^161–166^ Therefore, warfarin targeting an INR of 2.0–3.0 for at least 3 months postoperatively and up to 6 months is reasonable for patients undergoing bioprosthetic valve replacement and with low bleeding risk. After 3 to 6 months postoperatively, long-term treatment with daily aspirin 75–100 mg is recommended.		
New Recommendation	For patients undergoing mechanical valve replacement, if there is a history of IS or TIA before valve replacement, and the risk of bleeding is low, it is recommended to add aspirin to warfarin.	IIa	B
	Previous clinical studies have indicated that patients with a history of IS or TIA undergoing aortic mechanical valve replacement are at higher risk of thromboembolic complications. It is recommended to maintain the INR at a higher range (2.5–3.5) or add aspirin 75–100 mg daily.^167 168^		
New Recommendation	In patients with infective endocarditis complicated by IS or TIA, it is recommended that the decision regarding early surgery should be made jointly with the patient, neurologist, and cardiologist. Early surgery may provide benefits, especially if no evidence of intracranial haemorrhage or extensive neurological injury exists.	IIb	B
	A prospective cohort study evaluated the relationship between the timing of surgery and length of hospital stay with a 1 year mortality rate in 198 post-stroke patients, and found that early surgery was not associated with an increased risk of in-hospital mortality or a 1 year mortality rate.^169^		
New Recommendation	For patients with mechanical valves with a history of stroke or TIA, anticoagulation with dabigatran is not recommended.	III	B
	The RE-ALIGN trial compared dabigatran and warfarin to treat patients with mechanical heart valve replacement. The trial was terminated due to increased thromboembolic and bleeding events in the dabigatran group.^170^		
New Recommendation	For patients with IS or TIA complicated with left ventricular thrombus, starting anticoagulant therapy with warfarin for at least 3 months (INR range 2.0–3.0) is recommended to reduce the risk of stroke recurrence.	I	B
	In a meta-analysis of studies on thrombus after anterior myocardial infarction, vitamin K antagonists were found to reduce the incidence of stroke by 86% and achieve a left ventricular thrombus resolution rate of 68%.^171^		
New Recommendation	For patients with IS or TIA with a new left ventricular thrombus (<3 months), the efficacy and safety of direct oral anticoagulant therapy to reduce the risk of stroke recurrence is uncertain.	IIb	C
	A single-centre retrospective study showed that among 52 patients with left ventricular thrombus, 86% of patients had thrombus resolution after treatment with novel oral anticoagulants.^172^ However, due to the small sample size, no difference in the occurrence rate of embolic events was observed. Another large retrospective study analysed diagnosed left ventricular thrombus patients from three centres and compared 300 patients treated with warfarin to 185 patients treated with NOACs. The study found a higher incidence of stroke or systemic embolism in the group treated with novel oral anticoagulants (HR 2.71).^173 174^		
New Recommendation	In patients with acute anterior wall MI and an IS or TIA with reduced left ventricular ejection fraction (EF<50%), but no evidence of left ventricular thrombus, at least 3 months of oral anticoagulant therapy may be considered to reduce cardiogenic stroke recurrence.	IIb	C
	Patients with anterior wall MI and reduced ejection fraction are at increased risk of left ventricular thrombus formation.^172^ There is a lack of research on anticoagulant therapy in these patients. The recommendation for anticoagulation in this population is based on the current clinical practice and expert opinion.		
New Recommendation	For IS or TIA patients with left ventricular assist devices, it is reasonable to use warfarin and aspirin, and anticoagulant therapy with dabigatran is not recommended.	III	C
	Based on current clinical practice, the combination of warfarin and aspirin is the preferred approach for preventing recurrent IS in left ventricular assist device (LVAD) patients.^175^ The only RCT evaluating the benefits of dabigatran in LVAD implantation was terminated due to excessive thromboembolic events.^176^		
Reworded	The IS of unknown etiology and PFO should undergo appropriate and comprehensive evaluation to rule out other mechanisms of stroke. If a comprehensive evaluation suggests a potential causal relationship between PFO and IS, it is recommended that treatment decisions should involve discussions on PFO closure or medical treatment between the patient, neurologists, and cardiologists.	I	C
New Recommendation	For IS patients aged 18 to 60 years with PFO and no other identified cause after a comprehensive evaluation, if the PFO has high-risk anatomical features such as the atrial septal aneurysm or a significant right-to-left shunt, percutaneous closure of the PFO to prevent stroke recurrence is reasonable.	IIa	B
	Subgroup analyses of the RESPECT trial have shown significant benefits of PFO closure in patients with PFO accompanied by atrial septal aneurysm (HR 0.19, 95% CI 0.04 to 0.87, p=0.02) or substantial right-to-left shunting (HR 0.18, 95% CI 0.04 to 0.81, p=0.01).^177^ The results of the CLOSE trial (HR 0.03, 95% CI 0.00 to 0.26, p<0.001), the Gore REDUCE trial (HR 0.23, 95% CI 0.09 to 0.62, p=0.002), and the long-term follow-up of the RESPECT trial (HR 0.55, 95% CI 0.31 to 0.99, p=0.046) have all demonstrated significant clinical benefits of PFO closure over medical therapy in preventing stroke recurrence.^177–179^		
New Recommendation	For IS patients aged 18 to 60 years with PFO and no other identified cause after a comprehensive evaluation, if the PFO does not have high-risk anatomical features such as the atrial septal aneurysm or significant right-to-left shunt, the benefit of percutaneous closure of the PFO compared with antiplatelet therapy or warfarin treatment alone is still uncertain.	IIb	C
	The CLOSURE I, PC, and RESPECT trials did not demonstrate the superiority of PFO closure over medical therapy in unselected populations with PFO.^180–182^		
New Recommendation	For patients unsuitable for transcatheter occlusion of PFO, antiplatelet drugs such as aspirin or anticoagulant drugs (including warfarin and NOACs) should be selected according to the patient’s conditions.	IIa	C
	The current recommendation is based on clinical practice and expert opinion, as relevant trials are lacking.^183^		
New Recommendation	For patients with congenital heart disease and IS or TIA, anticoagulant treatment with warfarin is reasonable.	IIa	C
	Complex congenital heart disease is associated with an increased risk of stroke in adults.^184^ Based on current clinical practice and expert opinion, warfarin is considered reasonable if there are no contraindications to oral anticoagulant therapy.^185^		
New Recommendation	For patients with IS or TIA who also have Fontan palliation, anticoagulant treatment with warfarin is recommended to decrease stroke recurrence.	I	C
	The Fontan circulation and Fontan surgery could increase the risk of thromboembolic events through different pathophysiological mechanisms.^186^ Based on current clinical practice and expert opinion, warfarin is the preferred oral anticoagulant treatment option for patients with IS or TIA who also have Fontan palliation.		
New Recommendation	In patients with IS or TIA, if a cardiac tumour is located on the left side, surgical resection of the tumour can help reduce the risk of stroke recurrence.	IIa	C
	Currently, RCTs treating IS or TIA in patients with concomitant cardiac tumours are lacking.^187^ However, in a single-centre study, surgical resection of papillary fibroelastoma has been shown to reduce the risk of stroke.		
New Recommendation	In patients with coronary artery or peripheral arterial diseases, the efficacy and safety of low-dose NOACs combined with antiplatelets in reducing stroke recurrence may be effective.	IIb	C
	There are no specific study results regarding a novel antithrombotic regimen combining anticoagulation and antiplatelet therapy in the stroke population. However, the COMPASS trial found the combination of rivaroxaban 2.5 mg twice daily with aspirin 100 mg reduced stroke risk by nearly half without increasing the risk of haemorrhagic stroke in patients with coronary artery or peripheral artery disease (HR 0.51, 95% CI 0.38 to 0.68). A meta-analysis incorporating seven relevant RCTs, including patients with chronic coronary artery disease, heart failure, peripheral artery disease, and atrial fibrillation, found that low-dose novel oral anticoagulants combined with antiplatelet therapy can reduce the incidence of stroke. However, the difference was not statistically significant (IRR 0.73, 95% CI 0.53 to 1.01).^188 189^ The AXIOMATIC-SSP study investigated the efficacy and safety of low-dose novel oral anticoagulant combined with antiplatelet therapy to prevent recurrent stroke. The study is currently ongoing.^190^		

**Table:** 

8.3 small vessel stroke		COR	LOE
New Recommendation	For patients with small vessel stroke, the preventive effect of cilostazol might be beneficial.	IIb	B
	In the CSPS trial, which predominantly involved patients with lacunar stroke (74% of the participants), cilostazol was more effective than the placebo for secondary stroke prevention.^191^ In the CSPS II trial, compared with the aspirin group, the cilostazol group had a significantly reduced risk of the first occurrence of ischaemic or haemorrhagic stroke. The incidence of bleeding events was significantly reduced in the cilostazol group. However, subgroup analysis showed no significant reduction in recurrent stroke risk in participants with lacunar stroke.^192^		
Reworded	Indiscriminate long-term dual antiplatelet therapy is not recommended for patients with small vessel stroke.	IIa	B
New Recommendation	For patients with small vessel stroke, if tolerated, it is recommended to lower systolic blood pressure to below 130 mmHg and diastolic blood pressure to below 80 mmHg.	IIa	B
	The SPS3 trial enrolled 3020 patients with lacunar stroke and compared the effects of a systolic blood pressure target of<130 mmHg vs 130–149 mmHg on stroke recurrence and cognition. Although there was no statistically significant difference in stroke recurrence, the group with a systolic blood pressure target of<130 mmHg significantly reduced the incidence of cerebral haemorrhage.^193^		

**Table:** 

8.4 ischaemic stroke due to other etiologies		COR	LOE
Unchanged	In with IS or TIA caused by extracranial carotid or vertebral artery dissection, antithrombotic therapy should be used for at least 3–6 months to prevent stroke recurrence or TIA.	I	C
Unchanged	For patients with IS or TIA caused by extracranial carotid or vertebral artery dissection within 3 months of onset, it is reasonable to use antiplatelet or warfarin to prevent the recurrence of stroke or TIA.	IIa	B
New Recommendation	For patients with IS or TIA caused by an extracranial carotid artery or vertebral artery dissection, stenting may be considered if optimal medical treatment fails.	IIb	C
	Currently, RCTs to support the benefits of endovascular treatments in cervical artery dissection are lacking. However, some studies suggest a relatively low incidence of complications associated with endovascular treatments.^194 195^		
New Recommendation	Antiplatelet drugs are recommended for patients with IS or TIA caused by intracranial arterial dissection, but the risk of bleeding should be noted.	IIb	C
	High-quality RCTs for antithrombotic therapy for patients with intracranial artery dissection are lacking. However, a small retrospective study at a single centre suggested that anticoagulant therapy is safe in patients with intracranial artery dissection who do not have a concomitant subarachnoid haemorrhage (SAH).^196^ Given the risk of SAH associated with intracranial artery dissection and the lower bleeding risk of antiplatelet therapy, specifically aspirin, compared with anticoagulant therapy, it seems safer and more reasonable, based on current clinical practice and expert opinion, to administer aspirin to patients with intracranial artery dissection.^197 198^		
Revised	When patients with moyamoya disease have an IS or TIA, it is recommended to effectively manage the risk factors for stroke, and perform an individualised evaluation to select the appropriate timing and method for extracranial-intracranial arterial bypass.	IIa	C
	A meta-analysis and multicenter retrospective series have shown no difference between direct and indirect bypass surgery.^199 200^ An international survey conducted among renowned experts in moyamoya disease treatment reported that, compared with Asian respondents, most non-Asian respondents recommended antiplatelet therapy.^201^		
New Recommendation	For patients with moyamoya disease and IS or TIA, antiplatelet therapy with aspirin is recommended to reduce the risk of stroke recurrence. When aspirin is intolerable or ineffective, clopidogrel or other thienopyridine drugs can be selected. Long-term use of antiplatelet or dual antiplatelet increases the risk of bleeding.	IIb	C
	An international survey conducted among renowned experts in moyamoya disease treatment reported that, compared with Asian respondents, most non-Asian respondents recommended antiplatelet therapy.^201^ This recommendation is based on current clinical practice and expert opinion.		
New Recommendation	For patients with autoimmune vasculitis-related stroke, apart from treatment for the autoimmune disease, antiplatelet therapy is recommended, and a multidisciplinary team should manage patients.	IIa	C
	Currently, multiple clinical trials are focusing on evaluating the efficacy of immunotherapy in treating autoimmune vasculitis-related stroke.^202–210^ Based on current clinical practice and expert opinion, adding antiplatelet therapy to immunotherapy is recommended to treat autoimmune vasculitis-related stroke. It is crucial to manage this treatment with a multidisciplinary team.		
New Recommendation	For patients with IS related to infectious vasculitis and tumour vasculitis, antiplatelet or anticoagulant therapy are both reasonable according to the patient’s condition in addition to the treatment of the primary disease.	IIa	C
	Acyclovir is the preferred medication for the treatment of varicella-zoster virus.^211 212^ In patients diagnosed with neurosyphilis who present with a stroke, immediate administration of penicillin is recommended.^213^ Secondary stroke prevention in patients with HIV vasculopathy primarily focuses on daily antiplatelet therapy and restoring the immune system.^214 215^ Based on current clinical practice and expert opinion, the risk of stroke recurrence and treatment goals should be discussed with an infectious disease specialist.		
New Recommendation	For TIA or IS patients with hyperhomocysteinemia caused by genetic diseases, it is reasonable to use vitamin B12 and folic acid to reduce blood homocysteine levels.	I	C
	A study on the treatment of severe hyperhomocysteinemia in patients with cystathionine beta-synthase deficiency conducted in Australia, the Netherlands, and Ireland showed a significant reduction in the risk of vascular events compared with historical cohort studies (RR 0.091, 95% CI 0.043 to 0.190, p<0.001).^216^		
New Recommendation	For IS or TIA patients with Fabry disease, the efficacy of enzyme replacement therapy on stroke prevention is uncertain.	IIb	B
	An RCT in Fabry patients found that enzyme replacement therapy improved the pain-related quality of life.^217^ However, its impact on disease progression or mortality requires further investigation.		
New Recommendation	Patients with carotid webs who have experienced IS or TIA, without any other identifiable causes, are recommended to receive antiplatelet therapy to prevent recurrent stroke or TIA.	I	C
	The optimal medical treatment for symptomatic carotid webs remains unclear. Approximately 29% to 56% of patients with symptomatic carotid webs experience recurrent stroke.^218 219^ Carotid artery stenting or carotid endarterectomy are alternative treatment options for symptomatic carotid artery stenosis. A meta-analysis of 158 cases found that 56% of patients with medical treatment experienced recurrent stroke, while 72% of patients treated with percutaneous transluminal angioplasty did not experience recurrent stroke.^219^ In another prospective study, 16 patients were treated with stenting, and no recurrent strokes were reported.^220^		
New Recommendation	For patients who have carotid webs and experience a recurrent stroke, despite standard medical treatment, stenting may be considered.	IIb	C
	A meta-analysis involving 158 patients with carotid artery dissection indicated that 56% of patients treated with medication experienced recurrent strokes, while ultimately, 72% of patients underwent endovascular treatments (carotid artery stenting or carotid endarterectomy), and none of these patients experienced recurrent strokes.^218^ In another prospective study of 24 patients with stroke/ TIA caused by carotid artery dissection, 7 cases experienced recurrent strokes. Among them, 2 cases received dual antiplatelet therapy, 3 were on single-agent antiplatelet therapy, 1 received thrombolysis within 24 hours, and one did not receive antithrombotic treatment. In contrast, no recurrent strokes were observed among the 16 patients treated with stenting.^219^		
New Recommendation	For IS or TIA patients with fibromuscular dysplasia (FMD), without any other identifiable cause, antiplatelet therapy, blood pressure control, and lifestyle management are recommended to prevent stroke recurrence.	I	C
	In a registry study conducted in the United States, 73% of patients with FMD, received antiplatelet therapy, with aspirin being the most commonly used medication.^221 222^ There is no RCT comparing aspirin to placebo in patients with symptomatic or asymptomatic FMD. The recommendation to use antiplatelet therapy, blood pressure control, and lifestyle management as secondary prevention is based on current clinical practice and expert opinion.		
New Recommendation	For IS or TIA patients with FMD, in cases where recurrent strokes persist despite the administration of standard internal medical treatment, carotid artery angioplasty may be effective in the prevention of IS.	IIb	C
	A case series of 7 symptomatic patients with FMD showed no complications with balloon angioplasty.^223^ There is a lack of comparative data evaluating medical management vs endovascular treatments (such as angioplasty or stent placement) in patients with FMD and recurrent IS. Endovascular treatments are not recommended for asymptomatic FMD patients. In patients with recurrent strokes, despite optimal medical therapy, consideration may be given to endovascular treatments. The management of FMD-related arterial dissection and intracranial aneurysms follows similar principles of management as in those patient populations.		
New Recommendation	For patients with IS or TIA caused by FMD and arterial dissection, antiplatelet therapy can be used.	IIa	C
	In the United States fibromuscular dysplasia registry, it has been reported that 19% of patients with cervical artery dissection experience IS.^224^ There is a lack of high-quality studies specifically addressing the management of IS or TIA in patients with FMD complicated by arterial dissection. This recommendation is based on the expert opinion.		
New Recommendation	For patients with vertebrobasilar dolichoectasia and a history of IS or TIA with no other identifiable causes, antiplatelet or anticoagulant therapy is reasonable for preventing recurrent strokes.	Iia	B
	Currently, no RCTs compared antiplatelet therapy with conservative observation in the management of basilar artery dolichoectasia. However, compared with the natural history of the disease, antiplatelet therapy has been shown to reduce the risk of recurrent strokes.^225 226^		
New Recommendation	For patients with isolated positive anticardiolipin antibodies but who do not meet the diagnostic criteria for antiphospholipid syndrome (APS), and present with IS or TIA, it is recommended to use antiplatelet therapy alone to reduce the risk of stroke recurrence.	I	B
	In the subgroup analysis of the WARSS trial, individuals with a one-time positive antiphospholipid antibody did not experience a significant difference in stroke risk reduction when treated with warfarin (RR 0.99, 95% CI 0.75 to 1.13) or aspirin (RR 0.94, 95% CI 0.70 to 1.28).^227^		
New Recommendation	For patients with IS or TIA who meet the diagnostic criteria for antiphospholipid syndrome, in addition to the treatment of APS, it is recommended to choose warfarin to prevent recurrent thrombotic events.	IIa	C
	Currently, there are no specific antiplatelet trials for patients with IS or TIA who meet the diagnostic criteria for APS. The clinical expert consensus leans towards using warfarin, with a target INR of 2.0 to 3.0.^228–230^		
New Recommendation	The appropriate dose of warfarin is to maintain the INR between 2.0 and 3.0 to balance the therapeutic effect and bleeding risk for patients with IS or TIA who meet the diagnostic criteria for APS.	IIa	B
	Currently, there are no specific antiplatelet trials for patients with IS or TIA who meet the diagnostic criteria for APS. The clinical expert consensus leans towards using warfarin, with a target INR of 2.0 to 3.0.^228–230^		
New Recommendation	In patients diagnosed with IS or TIA, who also present with a concomitant APS characterised by a history of thrombosis and triple positive antiphospholipid antibodies, it has been observed that the use of rivaroxaban poses a greater risk of thrombotic events compared with warfarin. Therefore, it is not recommended to use rivaroxaban as a secondary prevention of thrombotic events.	III	B
	Multiple observational studies have shown an increased risk of arterial thrombosis and stroke recurrence with NOACs, especially in high-risk patients who are triple positive for antiphospholipid antibodies or have a history of arterial thrombosis.^231–233^ The ASTRO-APS trial included 48 patients with APS. After 1 year of follow-up, it was found that patients treated with apixaban had a higher incidence of stroke (6/23) compared with patients treated with warfarin (0/25). It suggests that apixaban may be less effective than warfarin for secondary stroke prevention in APS patients. However, the limited sample size and two protocol amendments in the trial limited the reliability of the conclusions drawn from this study.^233^		
New Recommendation	For IS or TIA patients complicated with cancer, after evaluating the benefits and risks, antiplatelet or anticoagulant therapy should be given based on the cancer type and stage, as well as the aetiology of the vascular event.	IIb	C
	Approximately 15% of cancer patients may experience stroke, with a high coagulable state being the most common cause of IS in cancer patients.^234^ The commonly used antithrombotic drugs for cancer patients with IS include low molecular weight heparin, warfarin, and NOACs. However, there is a lack of high-quality RCTs to support treatment.		
New Recommendation	For IS or TIA patients complicated with atrial fibrillation and cancer, in addition to actively treating the primary disease, consideration may be given to using NOACs instead of warfarin to prevent stroke recurrence.	IIb	B
	A meta-analysis, including three RCTs, a retrospective cohort study, and a case-control study, demonstrated that the use of NOACs in cancer patients with atrial fibrillation showed superior efficacy (in terms of stroke, systemic embolism, deep vein thrombosis, and all-cause mortality) and a higher level of safety (with regards to major organ bleeding) compared with warfarin.^235^		

**Table:** 

8.5 cryptogenic stroke		COR	LOE
New Recommendation	Aspirin is recommended as secondary prevention for patients with ESUS, and NOACs are not recommended.	III	B
	The NAVIGATE-ESUS trial randomly assigned 7213 patients to receive either 15 mg/day of rivaroxaban or 100 mg/day of aspirin. The results showed that there were 172 cases (4.8%) of stroke or systemic embolism in the rivaroxaban group compared with 160 cases (4.4%) in the aspirin group (5.1% vs 4.8%, p=0.52). However, rivaroxaban had a higher rate of major bleeding than aspirin (1.8% vs 0.7%, p<0.001).^236^ The RESPECT-ESUS trial randomised 5390 patients into two groups: the dabigatran group and the aspirin group. During a median follow-up of 19 months, there were 117 cases (6.6%) of recurrent stroke in the dabigatran group and 207 cases (7.7%) in the aspirin group (annualised rates of 4.1% vs 4.8%, p=0.10). The incidence of major bleeding was similar between the two groups (annualised rates of 1.7% vs 1.4%, p=0.30).^237^		

### Section 9: risk factor management and long-term intervention

**Figure 12:**
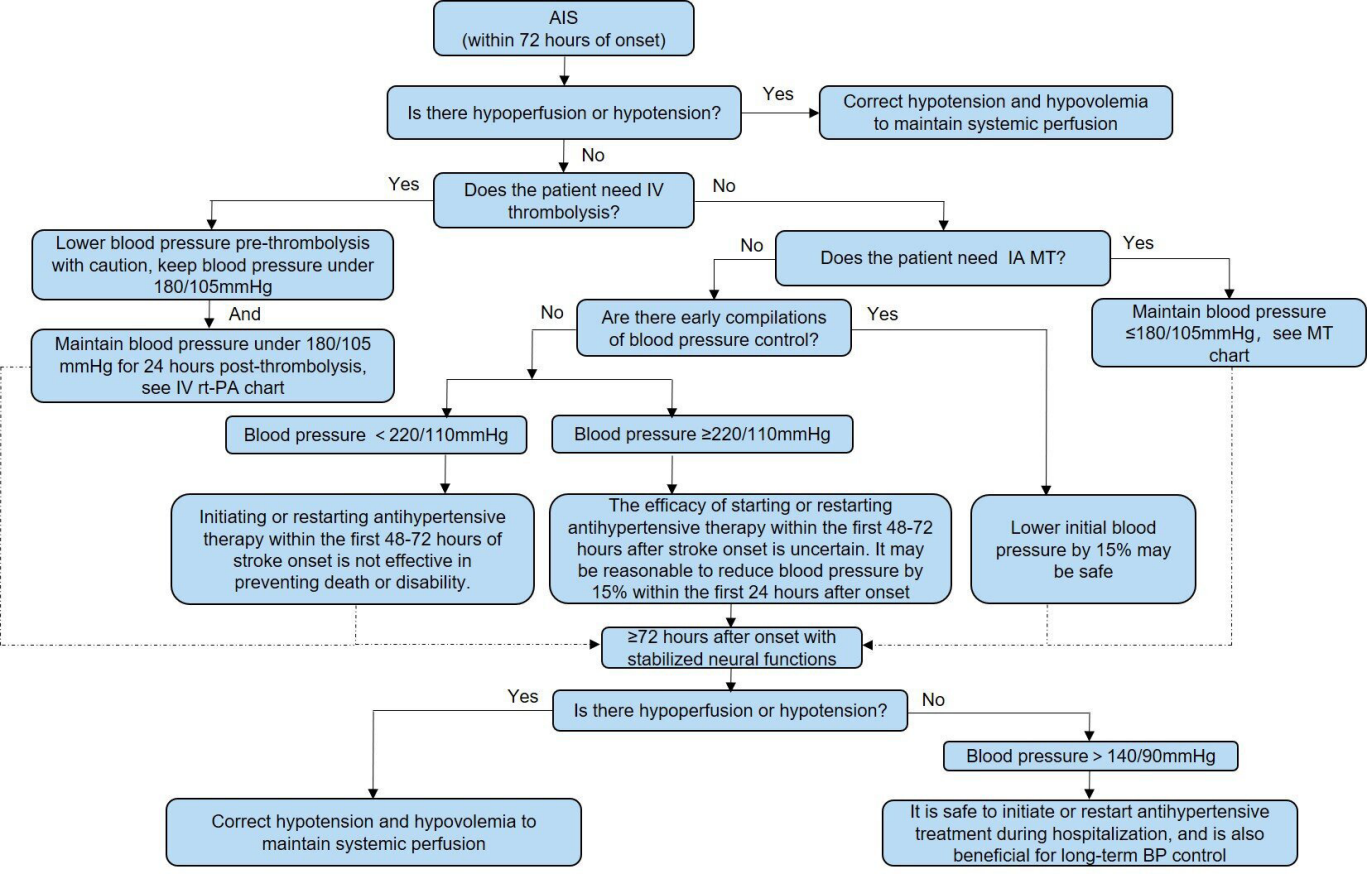
The blood pressure (BP) management within 72 hours after the onset of acute ischaemic stroke (AIS). IA, intra-arterial; IV, intravenous; MT, mechanical thrombectomy; rt-PA, recombinant tissue plasminogen activator.

**Figure 13:**
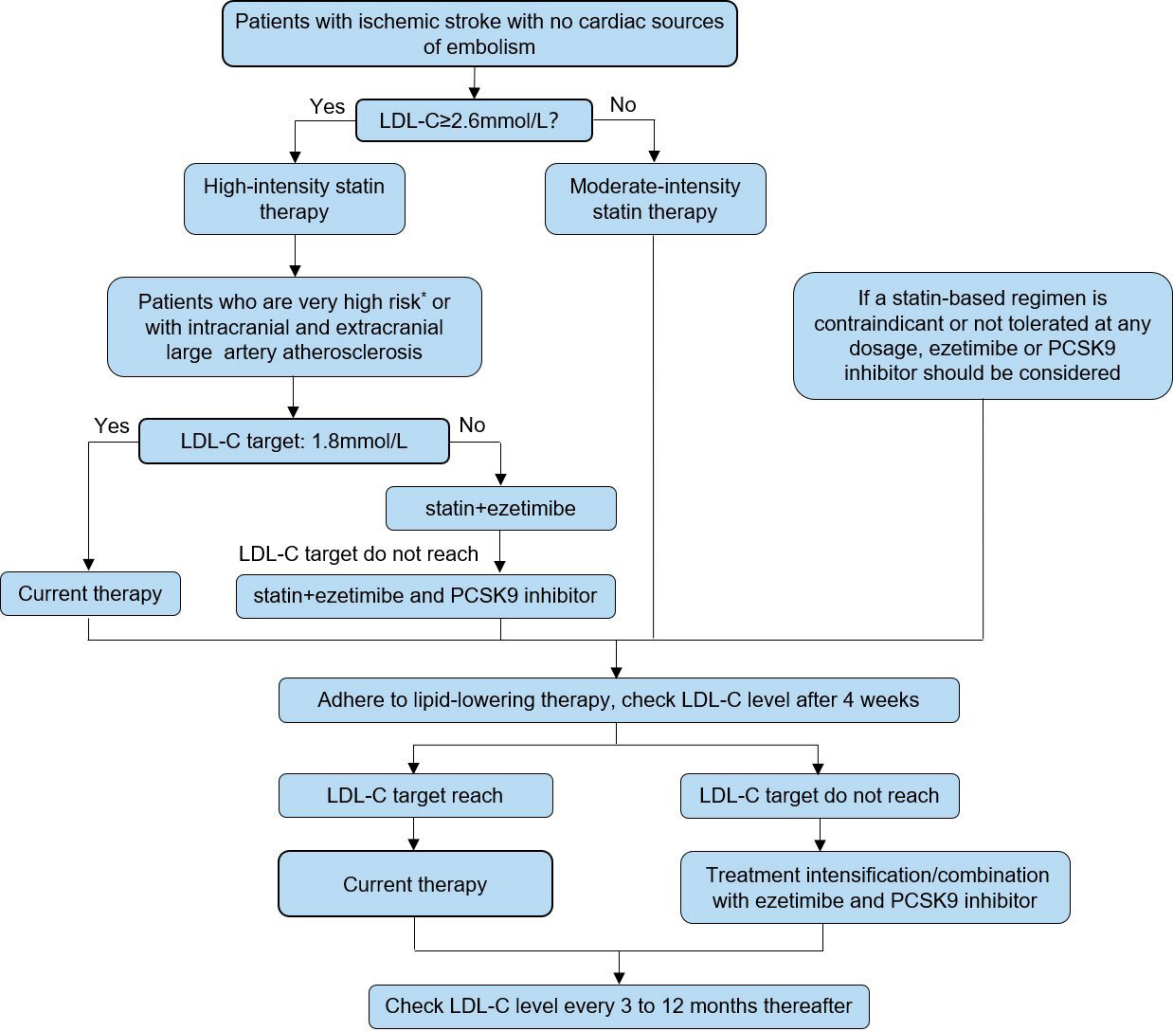
The lipid-lowering management in patients with acute ischaemic stroke. *Very high risk includes a history of multiple major ASCVD events or one major ASCVD event and multiple high-risk conditions. Major ASCVD events: history of ischaemic stroke; recent acute coronary syndrome (within the past 12 months); history of myocardial infarction (other than recent acute coronary syndrome event listed above); symptomatic peripheral arterial disease (history of claudication with ankle-brachial index <0.85 or previous revascularisation or amputation). High-risk conditions: age ≥65 years; heterozygous familial hypercholesterolaemia; history of coronary artery bypass surgery or percutaneous coronary intervention outside of the major ASCVD events; diabetes; hypertension; chronic kidney disease (estimated glomerular filtration rate, 15–59 mL/min/1.73 m^2^); current smoking. ASCVD, atherosclerotic cardiovascular disease; LDL-C, low-density lipoprotein cholesterol; PCSK9, proprotein convertase subtilisin/kexin 9.

**Figure 14:**
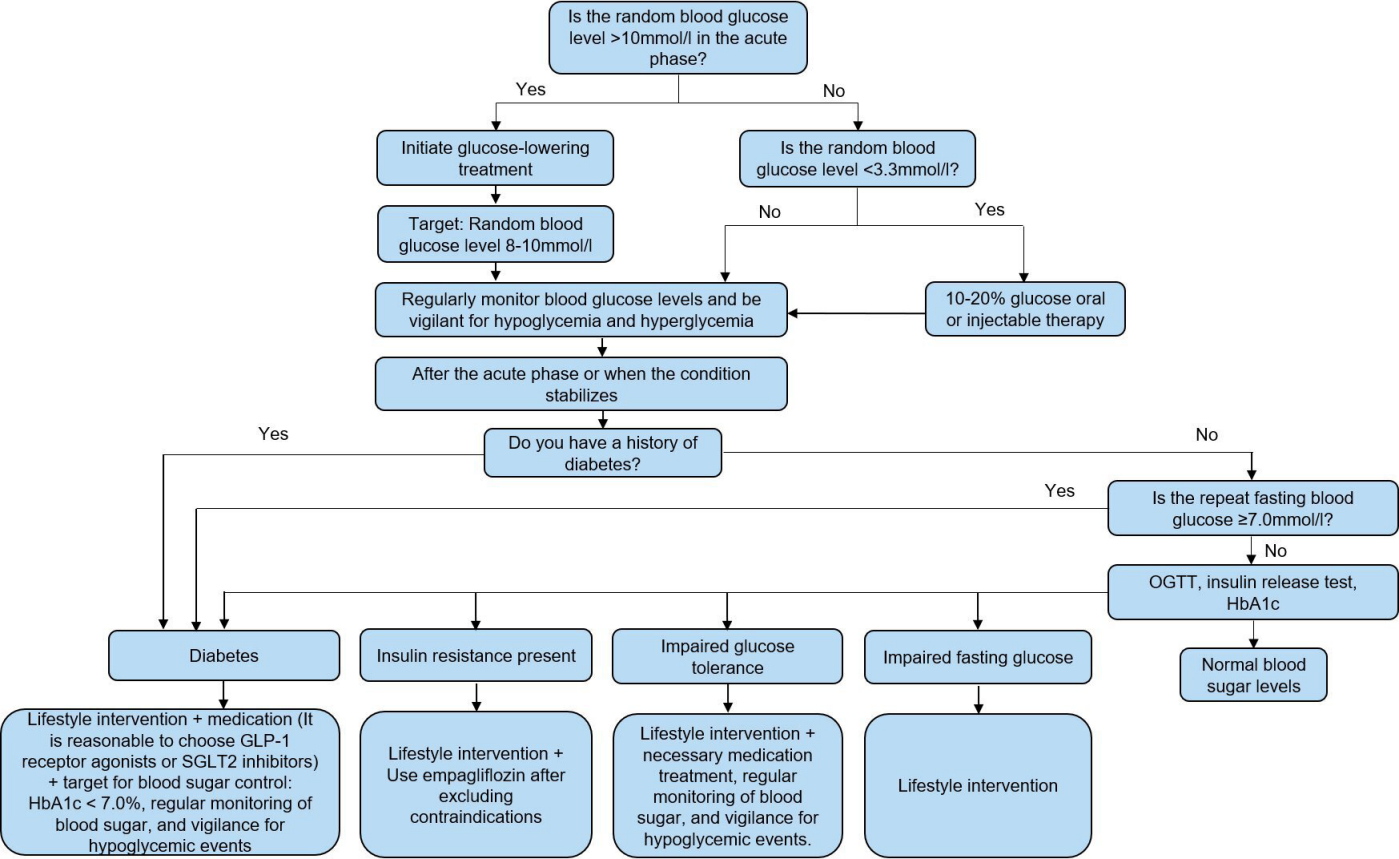
The flow chart for blood glucose management in patients with acute ischaemic stroke. GLP-1, glucagon-like peptide-1; HbA1c, glycated haemoglobin; OGTT, oral glucose tolerance test; SGLT2, sodium-glucose cotransporter 2.

The flow chart for blood pressure management within 72 hours after the onset of AIS is shown in figure 12. The flow chart for lipid-lowering management in patients with AIS is shown in figure 13. The flow chart for blood glucose management in patients with AIS is shown in figure 14.

#### 9.1 Blood pressure management

**Table:** 

9.1 Blood pressure management		COR	LOE
Reworded	For patients with a blood pressure <220/120 mm Hg, who have not received intravenous thrombolysis or IA MT and do not have any complications requiring urgent blood pressure reduction, initiating or restarting antihypertensive therapy within the first 48–72 hours after AIS showed no efficacy in preventing death or severe disability.	III	A
Reworded	For patients who have not received intravenous thrombolysis or IA MT, and have a blood pressure of ≥220/120 mm Hg without other complications requiring urgent blood pressure reduction, the efficacy of initiating or restarting antihypertensive therapy within the first 48–72 hours after AIS is uncertain. Lowering blood pressure by 15% within the first 24 hours after the onset of a stroke may be considered reasonable.	IIb	C
Reworded	For patients with AIS with concomitant comorbidities such as acute coronary events, acute heart failure, aortic dissection, haemorrhagic transformation after thrombolysis or pre-eclampsia/eclampsia, early antihypertensive therapy is indicated. An initial blood pressure reduction of 15% may be considered safe.	I	C
Reworded	Correction of hypotension and hypovolaemia is necessary after stroke to maintain adequate systemic perfusion and support the proper functioning of organs.	I	C
Reworded	For patients with AIS, the efficacy of pharmacologically induced hypertension is uncertain.	IIb	C
New recommendation	For blood pressure targets in patients who had a stroke, it is recommended lowering SBP below 130 mm Hg and DBP below 80 mm Hg, if tolerated by the patient.	I	B
	The SPS3 trial found that targeting an SBP of <130 mm Hg did not result in a significant reduction in stroke recurrence for patients with recent lacunar stroke, but the rate of ICH was reduced significantly (0.37, 95% CI 0.15 to 0.95, p=0.03).^238^ A recent meta-analysis showed a significant reduction in stroke with an intensive versus standard target (RR 0.78, 95% CI 0.64 to 0.96).^239^		
New recommendation	In patients with IS or TIA attributed to severe intracranial large artery stenosis (70-99%), it is recommended lowering SBP to below 140 mm Hg and DBP to below 90 mm Hg, if tolerated by the patient.	IIa	B
	The SAMMPRIS trial found that it is safe to control SBP within 140 mm Hg or lower in patients with IS or TIA attributed to severe intracranial large artery stenosis (70-99%), and this is associated with a lower risk of stroke recurrence.^240^		
New recommendation	There are insufficient data to provide specific guidance on the selection of antihypertensive medications following AIS. The choice of appropriate antihypertensive drugs should be based on individual patient considerations and the physician’s choice.	IIa	C
	A comprehensive meta-analysis that included three trials (PROGRESS, PRoFESS and PATS) aimed to evaluate the efficacy of ACE inhibitors (ACEI), angiotensin receptor blockers and diuretics in preventing stroke recurrence among Chinese patients with IS. The findings indicated that the type of medication did not influence the risk of stroke recurrence.^241^ Several clinical trials indicated that compared with ACEI, calcium channel blockers and placebo, beta-blockers may not significantly reduce the risk of stroke.^242–244^		

#### 9.2 Management of abnormal lipid metabolism

**Table:** 

9.2 Management of abnormal lipid metabolism		COR	LOE
New recommendation	For patients with non-cardioembolic IS or TIA with LDL-C levels ≥2.6 mmol/L (100 mg/dL), high-intensity statin therapy is recommended to reduce the risk of stroke recurrence.	I	A
	The SPARCL trial included adults with ischaemic or haemorrhagic stroke (or TIA, presumably owing to atherosclerotic causes) and an LDL-C level of 100–190 mg/dL.^245^ Eligible patients were randomised to atorvastatin 80 mg or placebo. The result revealed that in patients with recent stroke or TIA and without known coronary heart disease, 80 mg of atorvastatin per day reduced the overall incidence of strokes and cardiovascular events (atorvastatin group 11.2% vs placebo group 13.1%, adjusted HR 0.84, 95% CI 0.71 to 0.99). The secondary analysis of the SPARCL trial explored the effects of treatment in SPARCL subjects with type 2 diabetes mellitus or metabolic syndrome (MetS).^246^ This exploratory analysis found no difference in the effect of statin treatment in reducing these events in subjects with or without type 2 diabetes or MetS.		
New recommendation	High-intensity statin therapy is recommended for patients with non-cardioembolic IS or TIA with intracranial and extracranial atherosclerosis. If necessary, combination therapy with ezetimibe should be considered to achieve LDL-C levels below 1.8 mmol/L (70 mg/dL) or to reduce LDL-C levels by 50% or more, aiming to lower the risk of stroke and cardiovascular events.	I	A
	The TST trial included adults with cerebral infarction or high-risk TIA, and a clear indication for statin therapy. Eligible patients were randomly assigned to an LDL-C target of <70 mg/dL vs 90–110 mg/dL.^146^ To achieve the assigned LDL-C targets, statin therapy was intensified, and ezetimibe was added if necessary. Patients who achieved a target LDL-C level of less than 70 mg/dL had a lower risk of subsequent cardiovascular events compared with those who had a target range of 90–110 mg/dL (8.5% vs 10.9%, HR 0.78, 95% CI 0.61 to 0.98).		
Revised	For patients with IS or TIA, if their LDL-C levels remain above 1.8 mmol/L despite receiving maximum tolerated statin therapy, combination treatment with ezetimibe is recommended.	I	B
	The IMPROVE-IT trial involved adult patients with acute coronary syndrome to compare the cardiovascular risk of the combination of simvastatin and ezetimibe with simvastatin and placebo. The result indicated that when added to statin therapy, ezetimibe resulted in the incremental lowering of LDL-C levels and improved cardiovascular outcomes (HR 0.94, 95% CI 0.89 to 0.99).^247^		
Revised	For extremely high-risk patients who had an IS (stroke plus another major atherosclerotic cardiovascular disease (ASCVD) event or stroke plus multiple high-risk factors), if LDL-C levels remain above 1.8 mmol/L despite receiving maximum tolerated statin and ezetimibe combination therapy, the use of PCSK9 inhibitors is recommended to prevent ASCVD events.	IIa	B
	The FOURIER trial involved patients with ASCVD (including stroke) with LDL-C levels of 1.8 mmol/L or higher who received statin therapy.^248^ Patients were randomly assigned to receive evolocumab or a matching placebo. The result showed that relative to placebo, evolocumab treatment significantly reduced the risk of the primary endpoint (9.8% vs 11.3%, HR 0.85, 95% CI 0.79 to 0.92, p<0.001).		
New recommendation	For patients who cannot tolerate statins or have contraindications to statin therapy, the use of PCSK9 inhibitors or ezetimibe may be considered.	IIb	B
	The ODYSSEY ALTERNATIVE trial compared alirocumab with ezetimibe in patients at moderate to high cardiovascular risk with statin intolerance.^249^ The results showed that alirocumab reduced the mean LDL-C levels by 45.0% vs 14.6% with ezetimibe (mean difference 30.4%, p<0.001). Skeletal muscle-related events were less frequent with alirocumab versus atorvastatin (HR 0.61, 95% CI 0.38 to 0.99, p=0.042).		
New recommendation	For patients who had an IS or TIA with concurrent hypercholesterolaemia, the effectiveness of LDL-C-lowering medications and lifestyle adjustments should be assessed after 4–12 weeks of statin therapy based on the fasting lipid levels and safety indicators (liver transaminases and creatine kinase). Subsequently, medication adherence and safety should be monitored every 3–12 months as needed, considering medication adjustments.	I	A
	Lifestyle changes and statin therapy are often implemented together in the management of hypercholesterolaemia.^250^ The maximum percentage change in lipid levels typically occurs within 4–12 weeks after initiating statin therapy, at which time drug efficacy or initial adherence to therapy can be evaluated. The most frequent adverse effect of statin therapy is myopathy. Increased levels of liver enzymes may occur during statin therapy and are usually reversible. Periodical remeasurements of these indicators can confirm adherence to lipid-lowering therapy.^251^		
New recommendation	For patients who had an IS or TIA with fasting TG ≥135 mg/dL (1.52 mmol/L), who have received moderate or high-intensity statin therapy, an HbA1c level <10%, and no history of pancreatitis, atrial fibrillation, or severe heart failure, treatment with icosapent ethyl (2 g two times per day) can reduce the risk of stroke recurrence.	IIa	B
	The REDUCE-IT trial randomised 8179 patients with ASCVD, including a history of IS or TIA (70%) or diabetes with other risk factors (30%), to icosapent ethyl 2 g two times per day plus statin versus statin alone.^252^ Enrolment criteria included fasting TG of 135–499 mg/dL and LDL-C of 41–100 mg/dL on statin dose for ≥4 weeks. Over a median follow-up of 4.9 years, the trial revealed a 25% reduction (17.2% vs 22.0%, HR 0.75, 95% CI 0.68 to 0.83, p<0.001) in the primary endpoint of major adverse cardiovascular events with icosapent ethyl treatment compared with the control group. The JELIS trial involved Japanese patients with hypercholesterolaemia with serum total cholesterol of 6.5 mmol/L or higher.^253^ Patients with hypercholesterolaemia were randomly assigned to receive eicosapentaenoic acid (EPA) with statin (EPA group) or statin alone (no EPA group). In the secondary prevention subgroup, stroke occurred in 48 (10.5%) of 457 no EPA group and in 33 (6.8%) of 485 EPA group, showing a 20% relative reduction in recurrent stroke in the EPA group (HR 0.80, 95% CI 0.640 to 0.997).		

#### 9.3 Management of abnormal glucose metabolism

**Table:** 

9.3 Management of abnormal glucose metabolism		COR	LOE
Reworded	The prognosis of persistent hyperglycaemia in patients with AIS within 24 hours after onset is worse than that of normal blood glucose. Therefore, it is reasonable to treat hyperglycaemia by aiming for target blood glucose levels between 140 and 180 mg/dL (7.8–10.0 mmol/L), while closely monitoring to prevent hypoglycaemia.	IIa	C
Reworded	Hypoglycaemia (<60 mg/dL or 3.3 mmol/L) should be promptly corrected in patients with ischaemic cerebrovascular disease.	I	C
New recommendation	Diabetes, pre-diabetes and insulin resistance are independent risk factors for recurrent IS or death. Screening for the glucose metabolism status of patients who had a stroke should be emphasised.	IIa	B
	Multiple studies have found a correlation between pre-diabetes, insulin resistance, diabetes, and adverse outcomes such as the occurrence, recurrence, and mortality of IS.^254–257^ However, during the acute phase of stroke, there is a phenomenon of underdiagnosis for newly developed diabetes or pre-diabetes, which suggests that clinicians should prioritise screening for diabetes, pre-diabetes and insulin resistance in patients with IS or TIA.^258^		
New recommendation	For patients who had an IS or TIA with concomitant diabetes, the target for blood glucose control in the post-acute phase should be individualised. The effect of strict blood glucose control (eg, HbA1c ≤7%) on preventing stroke recurrence remains uncertain.	IIb	B
	Several international clinical trials have failed to demonstrate that intensified glycaemic control reduces the risk of macrovascular diseases or lowers the risk of all-cause mortality or stroke.^259–261^ Instead, there is a significant increase in the risk of severe hypoglycaemia. The ADVANCE study found that strict blood glucose control, aiming for HbA1c <6.5%, significantly reduces the occurrence of composite endpoints, including vascular events.^262^ Therefore, the overall recommendation is to target the level of HbA1c to ≤7%, but individualised adjustments should be made.		
Reworded	Individualised blood glucose control targets should be established. Hypoglycaemic events should be noted.	IIa	B
New recommendation	For patients who had an IS or TIA with pre-diabetes, lifestyle interventions such as a healthy diet, regular physical activity and smoking cessation are beneficial in preventing the progression of diabetes.	IIa	B
	Lifestyle intervention has emerged as a safe and effective approach to impede the progression of pre-diabetes toward diabetes. The Da Qing Diabetes Prevention Study, which followed participants for a period of 23 years, revealed that lifestyle intervention for patients with impaired glucose tolerance can significantly reduce the long-term risk of diabetes, cardiovascular events and mortality.^263^		
New recommendation	Metformin may be beneficial in preventing the progression of diabetes.	IIa	B
	The DPP study demonstrated the efficacy of both intensive lifestyle intervention and metformin in mitigating the progression from impaired glucose tolerance to diabetes.^264^ While intensive lifestyle intervention exhibits superior outcomes compared with metformin, it is noteworthy that metformin demonstrates favourable tolerability and cost-effectiveness.		
New recommendation	For patients who had an IS or TIA with diabetes, a combination of lifestyle interventions, nutritional support, self-management education and antihyperglycaemic medications is advised.	I	C
	Blood glucose management requires a multifaceted approach, including lifestyle modifications, nutritional support, diabetes self-education and glucose-lowering medications.^265 266^		
New recommendation	Newer antihyperglycaemic medications, such as glucagon-like peptide-1 receptor agonists and sodium-glucose cotransporter 2 inhibitors, which have demonstrated beneficial effects in reducing the risk of cardiovascular events, including stroke, MI and vascular mortality, may be considered viable options.	IIa	B
	The REWIND study included high-risk patients with type 2 diabetes and cardiovascular disease, randomly assigning them to receive either the dulaglutide or placebo. The primary composite endpoints included non-fatal MI, non-fatal stroke or vascular death. The study found a significant reduction in the risk of major composite endpoints with dulaglutide compared with placebo (HR 0.88, 95% CI 0.79 to 0.99).^267^ The CANVAS study found that the risk of vascular events and mortality in the canagliflozin group was significantly lower than in the placebo group (HR 0.86, 95% CI 0.75 to 0.97).^268^		
New recommendation	In patients without diabetes with recent IS or TIA and insulin resistance, after excluding contraindications, the use of pioglitazone may be beneficial in preventing recurrent strokes.	IIa	B
	In the IRIS trial, conducted among patients without diabetes with recent IS and insulin resistance, pioglitazone demonstrated a 24% reduction in the risk of stroke recurrence or MI compared with placebo (HR 0.76, 95% CI 0.62 to 0.93).^269^		

#### 9.4 Management of other risk factors

**Table:** 

9.4 Management of other risk factors		COR	LOE
Revised	Smoking cessation is recommended for patients who had an IS or TIA with a smoking history.	I	A
	Results from a prospective cohort study in China suggested that smoking increased the risk of recurrent stroke in patients with stroke and TIA.^270^ The Nanjing Stroke Registry Study showed that, after adjusting for major covariates, persistent smokers still had a higher likelihood of stroke recurrence when compared with non-smokers (HR 1.93, 95% CI 1.43 to 2.61).^271^ There was a strong dose–response relationship between the amount of smoking and the risk of recurrent stroke.^271^		
New recommendation	Regardless of smoking history, patients with IS or TIA should avoid exposure to smoking environments and passive smoking.	I	B
	Data obtained from the US National Health and Nutrition Examination Surveys have shown that high exposure to secondhand smoke was associated with higher odds of previous stroke (OR 1.46). There was a dose-dependent relationship between exposure to secondhand smoke and all-cause mortality after stroke.^272^		
Revised	For patients with IS or TIA who smoke, comprehensive smoking cessation measures, including medication and behavioural interventions, are recommended.	I	A
	Some meta-analyses and RCTs have shown that drug combination therapy is the most effective and safe way to quit smoking.^273–275^ In particular, standard doses of varenicline combined with nicotine replacement therapy significantly improve the rate of sustained smoking cessation.^273 274 276^		
Revised	For patients with IS or TIA who have not quit drinking, alcohol consumption should be moderate. A daily alcohol intake of ≤2 standard units for men and ≤1 standard unit for non-pregnant women may be considered reasonable.	IIa	B
	The EPIC-CVD study showed that the HRs of non-fatal stroke and fatal stroke (both ischaemic and haemorrhagic) were 1.04 (95% CI 1.02 to 1.07) and 1.05 (95% CI 0.98 to 1.13) with an increase of 12 g daily alcohol intake above the baseline alcohol intake (24 g/day for men and 10 g/day for women), respectively.^273^		
New recommendation	It is recommended that healthcare professionals screen exercise capacity in patients who had a chronic IS with movement disorders, formulate personalised exercise plans and provide supervision.	I	B
	Currently, most of the evidence for physical activity in the secondary prevention of stroke comes from patients with incomplete loss of motor capacity.^277^ For patients unable to engage in regular physical activity, individualised exercise programmes should be developed based on their exercise endurance, stage of recovery, environment, available social support and exercise preferences.^278^ Some preliminary trial results suggest that aerobic exercise after the acute phase can improve cardiovascular health and reduce cardiovascular disease risk after adequate pre-exercise screening in patients with movement disorders.^279^		
New recommendation	For patients who had an IS or TIA with a good functional capacity, it is recommended engaging in moderate-intensity exercises, such as brisk walking, for at least three to four times per week (10 min/session), or aerobic exercises like brisk walking or jogging for at least two times per week (20 min/session) after the acute phase.	I	B
	The 3-year follow-up data from 227 patients in the drug treatment group of the SAMMPRIS trial showed that the risk of IS recurrence was 6.7 times higher for those with substandard physical activity levels (no or no regular moderate exercise (brisk walking or slow cycling ≥10 min) or vigorous exercise (jogging or brisk cycling ≥20 min)).^240^ More physical activity was associated with a 40% reduction in the risk of composite endpoint events (IS, MI, vascular death).		
New recommendation	Aerobic exercise is not recommended for patients who had a subacute IS with moderate severity (NIHSS score of 5–12).	III	B
	The PHYS-STROKE study found that for patients who had a stroke with NIHSS scores of 5–12, combined aerobic exercise with standard rehabilitation treatment increased the risk of stroke recurrence.^280 281^		
New recommendation	In overweight or obese patients with IS or TIA, weight reduction can reduce the risk of ASCVD.	I	B
	Currently, some RCTs which involve patients with diabetes have shown that weight control could significantly reduce SBP, glucose, TG and the incidence of cardiovascular events.^281–283^		
New recommendation	For obese patients with IS or TIA, multiple lifestyle adjustments or behaviour strategies should be employed based on individual circumstances to achieve the goals of weight management.	I	B
	A meta-analysis involving 122 RCTs and 2 observational studies showed that lifestyle adjustment and behavioural intervention could help lose weight safely and effectively.^284^		
New recommendation	For patients with IS or TIA, it is recommended following a diet with appropriate calorie and nutrient intake, which includes increased consumption of whole grains, legumes, fruits, vegetables and low-fat dairy products while decreasing the intake of saturated and trans fats. Additionally, there should be a moderate reduction in sodium intake and an increase in potassium intake. The practical use of potassium-containing salt substitutes is encouraged as it can contribute to lowering blood pressure and reducing the risk of stroke recurrence.	I	B
	Cohort studies have shown that increased intake of nuts, olive oil and fruits can reduce stroke risk by 28%, 31% and 52.3%.^270 285^ One RCT study from China confirmed that reducing sodium intake and increasing potassium intake also reduced stroke risk (HR 0.86, 95% CI 0.77 to 0.96, p=0.006).^286^		
New recommendation	Assessing nutritional risk promptly upon hospital admission is recommended for patients who had an IS or TIA. Individualised nutrition plans with targeted interventions should be implemented for patients identified with nutritional risk, along with regular screening.	IIb	B
	The study based on the Third China National Stroke Registry data found that moderate-to-severe malnutrition risk was associated with an increased risk of long-term death and major disability in patients with IS (OR 2.25, 95% CI 1.75 to 2.90, for controlling nutritional status score; OR 2.10, 95% CI 1.63 to 2.69, for geriatric nutritional risk index; OR 3.36, 95% CI 2.33 to 4.84, for prognostic nutritional index).^287^ The EFFORT study found that compared with standard hospital diets, individualised nutritional support therapy reduced the risk of 30-day adverse endpoint events by 21% (OR 0.79, 95% CI 0.64 to 0.97) and death by 35% (OR 0.65, 95% CI 0.47 to 0.91).^288^		
Unchanged	Routine screening for obstructive sleep apnoea in patients with recent IS is not recommended.	III	B
Revised	The association between oral contraceptives and stroke needs further confirmation through prospective studies. Oral contraceptives may be linked to various types of strokes, especially in patients with hypertension. Long-term and high-dose use of oral contraceptives is not recommended, particularly in individuals with hypertension.	III	B
	A study examined the association between self-reported oral contraceptive and hormone replacement therapy use and stroke risk in 257 194 women from the UK Biobank, and found an increased rate of any stroke (HR 2.49, 95% CI 1.44 to 4.30) and IS (HR 1.93, 95% CI 1.05 to 3.57).^289^ A dose–response meta-analysis involving 6 cohort studies and 12 case–control studies showed that longer and higher doses of oral hormonal contraceptives were associated with an increased risk of IS, with ORs 1.24 (95% CI 1.04 to 1.49) and 1.20 (95% CI 1.17 to 1.22), respectively.^290^		
Reworded	The relationship between drug use and stroke requires further investigation. Acute drug use in the previous 24 hours may be a risk factor for stroke occurrence and poor prognosis.	III	C
Reworded	For patients with recent IS or TIA and mild-to-moderate homocysteine elevation, supplementation with folate, vitamin B_6_ and vitamin B_12_ could effectively lower homocysteine levels. There is insufficient evidence to support homocysteine level reduction as a strategy for reducing the risk of stroke recurrence.	IIb	B
